# Combined Culture-Based and Culture-Independent Approaches Provide Insights into Diversity of Jakobids, an Extremely Plesiomorphic Eukaryotic Lineage

**DOI:** 10.3389/fmicb.2015.01288

**Published:** 2015-11-18

**Authors:** Tomáš Pánek, Petr Táborský, Maria G. Pachiadaki, Miluše Hroudová, Čestmír Vlček, Virginia P. Edgcomb, Ivan Čepička

**Affiliations:** ^1^Department of Zoology, Faculty of Science, Charles University in PraguePrague, Czech Republic; ^2^Geology and Geophysics Department, Woods Hole Oceanographic InstitutionWoods Hole, MA, USA; ^3^Department of Genomics and Bioinformatics, Institute of Molecular Genetics, Czech Academy of SciencesPrague, Czech Republic

**Keywords:** cryptic species, environmental clones, marine communities, species diversity, anaerobic protists

## Abstract

We used culture-based and culture-independent approaches to discover diversity and ecology of anaerobic jakobids (Excavata: Jakobida), an overlooked, deep-branching lineage of free-living nanoflagellates related to Euglenozoa. Jakobids are among a few lineages of nanoflagellates frequently detected in anoxic habitats by PCR-based studies, however only two strains of a single jakobid species have been isolated from those habitats. We recovered 712 environmental sequences and cultured 21 new isolates of anaerobic jakobids that collectively represent at least ten different species in total, from which four are uncultured. Two cultured species have never been detected by environmental, PCR-based methods. Surprisingly, culture-based and culture-independent approaches were able to reveal a relatively high proportion of overall species diversity of anaerobic jakobids—60 or 80%, respectively. Our phylogenetic analyses based on SSU rDNA and six protein-coding genes showed that anaerobic jakobids constitute a clade of morphologically similar, but genetically and ecologically diverse protists—*Stygiellidae* fam. nov. Our investigation combines culture-based and environmental molecular-based approaches to capture a wider extent of species diversity and shows Stygiellidae as a group that ordinarily inhabits anoxic, sulfide- and ammonium-rich marine habitats worldwide.

## Introduction

Decreasing sequencing costs have contributed to the generation of huge amounts of new genetic information from environmental samples that are providing novel insight into eukaryotic diversity and revealing diverse communities of protists in virtually all types of habitats including “extreme” ones (e.g., Amaral Zettler et al., 2002; Alexander et al., 2009; Edgcomb et al., 2011a,b). The most commonly used marker in such PCR-based environmental studies is the small subunit ribosomal RNA gene (SSU rDNA) or its hypervariable regions V4 and V9 (Pawlowski et al., 2012; Guillou et al., 2013; Lie et al., 2014; Massana et al., 2015; de Vargas et al., 2015). Using this marker, environmental studies indicated that the real species diversity of protists may be orders of magnitude greater than previously thought (Pawlowski et al., 2012). However, observed genetic diversity is not easily transferable into a precise number of species, because divergence within a single molecular marker may not correspond with an equivalent level of phenotypic differentiation among organisms (Lowe et al., 2005; Logares et al., 2007). Furthermore, the level of both intra- and interspecific sequence variability may differ significantly between lineages (Caron et al., 2009). Conversely, morphological criteria are often insufficient for distinguishing protistan species, and a single morphospecies may encompass huge cryptic species diversity (Weisse, 2008). Thus, the diversity of free-living eukaryotic microorganisms is a subject of general debate.

Studies combining culture-independent and culture-based approaches (e.g., Berney et al., 2013) have value for environmental data interpretation and reveal different types of potential artifacts. However, the number of studies combining both approaches is still very low. Additionally, little is known about the biogeography and interactions of individual protistan lineages in extreme and non-canonical environments including anoxic sites (Stock et al., 2013).

Environmental PCR-based studies indicate that a relatively low number of protistan phylotypes are abundant and cosmopolitan. For example, just 0.35% of OTUs reported from photic zone plankton communities represents hyper-dominant cosmopolitan taxa. The fact that only 2–17 OTUs (0.2–8% of total OTUs in the sample) dominated each sample in this environment indicates that just a small proportion of eukaryotic taxa play a major role (in terms of abundance) at any one time for local ecosystem function (de Vargas et al., 2015).

Because many uncultured protistan lineages detected in environmental studies are represented by only a single or a few sequences, the existence of an extremely diverse protistan “rare biosphere” was proposed (Caron and Countway, 2009). The term “rare biosphere” should not be considered simply as a synonym for assemblage of rare species. It is instead the assemblage of species that are rare in the original community (in dormant or active stage) at the time of sampling. It was shown that microbial members of the rare biosphere can become abundant in a community following some sort of disturbance (Sjöstedt et al., 2012). Thus, the composition of the rare biosphere is probably changing over time and its members can have important roles in maintaining ecosystem processes under different environmental conditions (Caron et al., 2012; Sjöstedt et al., 2012).

In this study, we have focused on the diversity and ecological role of Jakobida, an understudied but important lineage of the supergroup Excavata. Excavates are rarely detected in environmental sequence libraries and only approximately 2,500 species have been described so far (see Adl et al., 2007). This group includes several plesiomorphic lineages [i.e., *Carpediemonas*-like lineages (CLOs), trimastigids, and jakobids] which are striking examples of the species poverty of this group, because they are known based on only a few species and several strains. Furthermore, jakobids are probably a crucial lineage for our understanding the evolution of Excavata and possibly the whole of Eukaryota.

Jakobids are heterotrophic biflagellates. They constitute a deep-branching clade within Excavata and are divided into two lineages, Andalucina and Histionina. In total, they are comprised of only nine described species, the majority of which have been reported from fresh water; two species, *Jakoba libera* and *Andalucia incarcerata* (= *Stygiella incarcerata* comb. nov.) live in marine environments. The latter species is the only described anaerobic jakobid.

Although jakobids were not recognized as a taxon until the 1990s, and their diversity remains understudied, they have recently attracted considerable attention because of their plesiomorphic, bacterial-like mitochondrial genomes (Burger et al., 2013). In addition, jakobid cells possess a plesiomorphic arrangement and composition of the flagellar apparatus (Simpson and Patterson, 2001; Yubuki and Leander, 2013).

Recently, Derelle et al. (2015) proposed that the root of the eukaryotic tree lies between the Opimoda and Diphoda groups, and the last common ancestor of all eukaryotes was probably a jakobid-like protist. Derelle et al. showed that malawimonads, a small group of heterotrophic nanoflagellates that are almost indistinguishable from jakobids by light and electron microscopy (O'Kelly and Nerad, 1999), are not closely related to other Excavata. Instead, they form a clan with Amorphea (Opimoda), whereas jakobids and other excavates belong to Diphoda.

Although the jakobids have been frequently detected in anoxic habitats by environmental approaches, only two strains of a single species have been cultured so far. A comprehensive phylogenetic analysis including environmental sequences closely related to jakobids has been missing, and the monophyly of jakobids detected in anoxic/microoxic habitats has been unclear (Simpson et al., 2008). We cultured 21 new jakobid strains from various marine anoxic/microoxic habitats worldwide. Subsequently, we compared data from the strains with data obtained from environmental studies. Our results show that anaerobic jakobids constitute a globally distributed clade and are relatively common in anoxic marine environments.

## Materials and methods

### Organisms

As detailed in the Supplementary Material S1, most of the 21 strains were isolated from marine/brackish coastal sediments; strain LUC3N was obtained from sediments 20 m below the sea surface (see S1 for details). Samples were initially inoculated into the artificial seawater-based ATCC medium 1525 and then subcultured once a week.

### Salinity ranges for growth

Five different cerophyll-based media (see Supplementary Material S1) with salinities ranging from freshwater to 74 ppt were prepared to determine salinities suitable for growth of monoeukaryotic cultures. 0.25 ml of the culture was used for transfers into all types of media and cultures were examined after 4 and 7 days. Active growth at a particular salinity was confirmed by a transfer into a fresh medium with the same salinity. In order to reduce stress caused by sharp changes in salinity, we established cultures with extreme salinities by inoculation of actively growing cells from cultures with 19 and 56 ppt salinity. All experiments were done in triplicate.

### Light microscopy

Morphological observations were performed using a BX51TF Microscope equipped with a DP70 camera (Olympus). Living cells were observed using Differential Interference Contrast. Protargol-stained preparations were prepared following Nie's (1950) protocol as modified by Pánek et al. (2014a) and observed by bright-field microscopy. Cell length was measured for 50 cells for each isolate.

### Transmission electron microscopy

The cell suspension of strain LUC3N with addition of 20% BSA was frozen using the high-pressure freezer (Leica EM Pact II) and then transferred to the freeze substitution unit (Leica EM AFS). The ice in the specimen was replaced by anhydrous acetone containing 2% osmium tetroxide. The sample was embedded in EMbed-812 (EMS) and polymerized at 62°C for 48 h. The ultrathin sections were stained with uranyl acetate (2%) and lead citrate and examined using a TEM JEOL 1011.

### Nucleic acid extraction, PCR amplification, sanger and 454 sequencing

SSU rDNA was amplified from genomic DNA using universal eukaryotic primers (Medlin et al., 1988); the alpha-tubulin gene of strains LUC3N and PC1 and beta-tubulin gene of strain LUC3N were amplified using universal eukaryotic primers as described in Edgcomb et al. (2001) and Yoon et al. (2008). For details of methods for nucleic acid extraction, PCR amplification, cloning and sequencing see Supplementary Material S1. Total RNA was extracted from a monoeukaryotic culture of strain LUC3N. Methods used for cDNA library construction, 454 sequencing, cluster assembly, and gene transcript annotation for LUC3N are described in the Supplementary Material S1. Sequences reported in this study are available in GenBank (KP144389–KP144409). Sequences of other four protein-coding genes from *Velundella trypanoides* (454 contigs) are deposited as Supplementary Material S8 (cytosolic HSP70, HSP90, EF1A, EF2).

### SSU rDNA dataset construction

**Figure 1:**
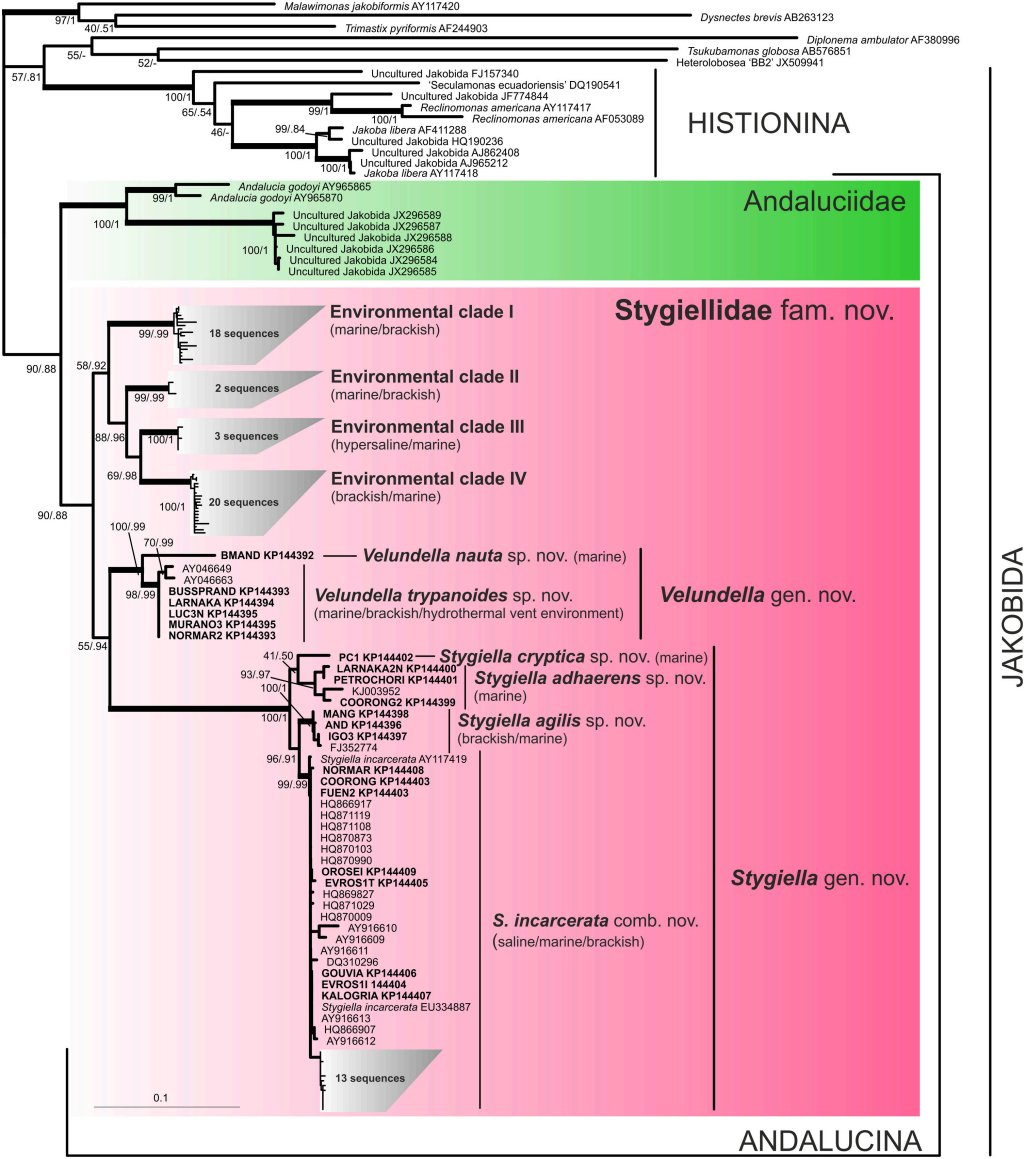
**Phylogenetic tree of Jakobida based on small subunit ribosomal rDNA**. The tree is based on alignment of 1588 nucleotide positions and 123 OTUs. The topology was constructed in RAxML using maximum likelihood (GTRGAMMAI model). The values at nodes represent RAxML bootstraps/PhyloBayes posterior probabilities. The values lower than 50% or 0.5 are marked by “−”. Clades supported by bootstrap/posterior probability higher than 95/0.95 are marked by thick branches. Sequences from newly isolated strains are in bold. Environmental sequences are represented by their GenBank accession numbers only.

A final dataset of SSU rDNA genes containing sequences from newly reported strains and cultured and uncultured jakobids from GenBank was constructed as described in the Supplementary Material S1. The final analysis included sequences at least 490 bp in length (see Figure 1). For the phylogenetic tree containing Sanger clone sequences shorter than 490 bp, see Supplementary Material S2, Figure S2.5. Datasets were aligned using the MAFFT 7.110 server (*http://mafft.cbrc.jp/alignment/server/*) with the G-INS-i algorithm (Katoh et al., 2005). The alignment was manually edited in BioEdit 7.0.4.1 (Hall, 1999) and is deposited in the Supplementary Material S3.

### Construction of protein datasets

Selected translated amino acid sequences from LUC3N were added to single-protein datasets constructed as described in the Supplementary Material S1. Amino acid sequences were aligned using the MAFFT 7.110 server at default settings and trimmed manually. To test for paralogs or contaminants, we performed phylogenetic analyses of the alignments (not shown) and checked the trees manually. A final multi-protein dataset (deposited in the Supplementary Material S4) was constructed by concatenation of independently aligned single-protein datasets for actin, β-tubulin, cytosolic HSP70, cytosolic HSP90, EF-2, and EF-1α genes. Gene sequences for α-tubulin were analyzed separately. A list of protein sequences used in our phylogenetic analyses is deposited in the Supplementary Material S5.

### Phylogenetic analyses

Phylogenetic trees were constructed in RAxML 8.0.19 (Stamatakis, 2006) using GTRGAMMAI (nucleotides) or PROTGAMMAILG (amino acid residues) models with 100 maximum likelihood tree searches. Branch support was estimated from 1000 non-parametric bootstrap replicates. PhyloBayes 3.3 (Lartillot and Philippe, 2004) was run on all datasets using CAT GTR (nucleotides) or CAT POI model (amino acid residues). Two or four independent chains were run until their maximum observed discrepancy was < 0.1, and the effective sample size of all model characteristics was at least 100. Consensus trees and posterior probabilities were than calculated using the BP comp program with 25% of generations discarded as burn-in, and sampling every 10 trees.

### Data mining in pyrotag archives

To find jakobid sequences in environmental pyrotag archives, we used the QIIME package 1.7 (Caporaso et al., 2010). Sequences from each dataset were clustered using a 97% cut-off and representative sequences of each OTU_97_ were taxonomically annotated against both the Silva 111 (Quast et al., 2013) and the PR2-119 databases (Guillou et al., 2013).

### Genetic distances

We computed uncorrected p distances among and within particular isolates using the region corresponding to positions 545–1544 (SSU rDNA) and 1645–1774 (V9) in KP144395 sequence.

## Results

### General morphology of cultured strains

We established protist cultures from oxygen-poor localities, predominantly from freshwater and marine littoral sediments. Jakobids were present in 21 of ~200 marine/brackish cultures and were never observed in freshwater cultures (~250 cultures). The cells slowly died when exposed to oxygen, suggesting they were anaerobic or microaerophilic. Accordingly, strains LUC3N, BUSSPRAND, and PC1 were successfully grown in an anaerobic chamber for a long period (>150 days).

The cells morphologically resembled other naked jakobids and formed two morphotypes previously reported in a survey of *Stygiella incarcerata* by Simpson and Patterson (2001): (a) frequently attaching cells with a conspicuous groove (grooved cells) and (b) cells with a less distinct, shortened or narrower groove (swimming cells). Both morphotypes were able to move with a spiraling motion; the swimming cells moved rapidly and usually did not attach to the substrate. Examination of the morphology was complicated due to the variability among cells within a single strain combined with similarity of swimming and grooved cells among strains. Thus, we had to investigate a lot of cells from several culture passages to distinguish between species properly. Dimensions of living and protargol-stained specimens of grooved cells of all species are documented in the Supplementary Material S2, Table S2.1.

### Phylogeny of anaerobic jakobids

A comprehensive search of sequence data in GenBank revealed 83 environmental SSU rDNA clones of jakobids from a variety of marine, hypersaline, and brackish oxygen-poor waters and sediments worldwide (see Supplementary Material S6). The phylogenetic position of the new strains and environmental clones was determined using maximum likelihood and Bayesian methods (Figure 1). Similar to previous analyses, jakobids were not recovered as a clade and formed two lineages (Lara et al., 2006; Simpson et al., 2008). The first one, Histionina *sensu* Cavalier-Smith (2013), was highly supported. The second lineage, Andalucina *sensu* Cavalier-Smith (2013), was less supported and contained *Andalucia godoyi, Stygiella incarcerata* as well as sequences from 21 new jakobid strains, all environmental clones of jakobids from oxygen poor sites and six clones from the soda lake Nakuru.

Andalucina split into two clades, the first clade, Andaluciidae, contained aerobic *Andalucia godoyi* and six environmental clones of an uncultured lineage from the lake Nakuru. The second clade, Stygiellidae fam. nov. included all new isolates of anaerobic jakobids, two previously published strains of *Stygiella incarcerata*, and environmental clones of jakobids from oxygen-poor sites. SSU rDNA sequences of Andalucina, including environmental lineages, contained the specific C:G base pair within the basal stem of helix 27 as described by Lara et al. (2006); the other jakobids displayed an A:T base pair in the same position.

Stygiellidae split into six statistically well-supported lineages: *Stygiella* gen. nov., *Velundella* gen. nov., and four environmental clades (EC I–IV). The interrelationships among these lineages remained unresolved except for EC II–IV which formed a relatively well-supported clade. Genetic distances among SSU rDNA sequences of Stygiellidae are documented in the Supplementary Material S7. Monophyly of particular *Stygiella* and *Velundella* species as well as EC I–IV was statistically highly supported; *Stygiella cryptica* sp. nov. and *Velundella nauta* sp. nov. were represented by only a single sequence.

**Figure 2:**
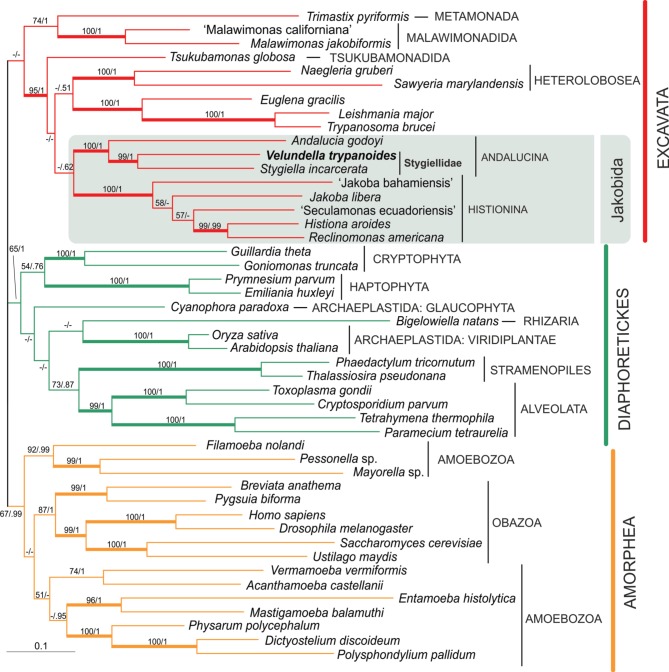
**Phylogenetic tree of eukaryotes based on concatenation of six protein-coding genes: actin, β-tubulin, EF1α, EF2, HSP70, HSP90**. The tree is based on alignment of 3200 amino acid positions and 47 taxa. The topology was constructed in RAxML using maximum likelihood (PROTGAMMAILG model with four partitions). PhyloBayes was run under CAT POI model. The values at nodes represent RAxML bootstraps/PhyloBayes posterior probabilities. The values lower than 50% or 0.5 are marked by “−”. Clades supported by bootstrap/posterior probability higher than 95/0.95 are marked by thick branches.

In order to pinpoint relationships among the genera *Velundella, Stygiella*, and *Andalucia* more clearly, we carried out a concatenated analysis of six protein-coding genes (Figure 2). Both Andalucina and Histionina appeared robustly monophyletic; *Velundella* and *Stygiella* constituted a robust clade.

The alpha-tubulin gene was not included in the final multi-protein analysis, because Andalucina and Histionina contain strikingly different sequences that possibly represent different paralogs (Simpson et al., 2008). Our phylogenetic analysis is consistent with this assumption since the newly obtained sequences of *Velundella trypanoides* sp. nov. and *Stygiella cryptica* grouped with other members of Andalucina, Trichozoa and opisthokonts; specifically with *S. incarcerata* and *Andalucia godoyi* (see Supplementary Material S2, Figure S2.7.).

### *Stygiella* gen. nov.

#### For extended version, see Supplementary Material S2, chapter 1.1.

**Figure 3:**
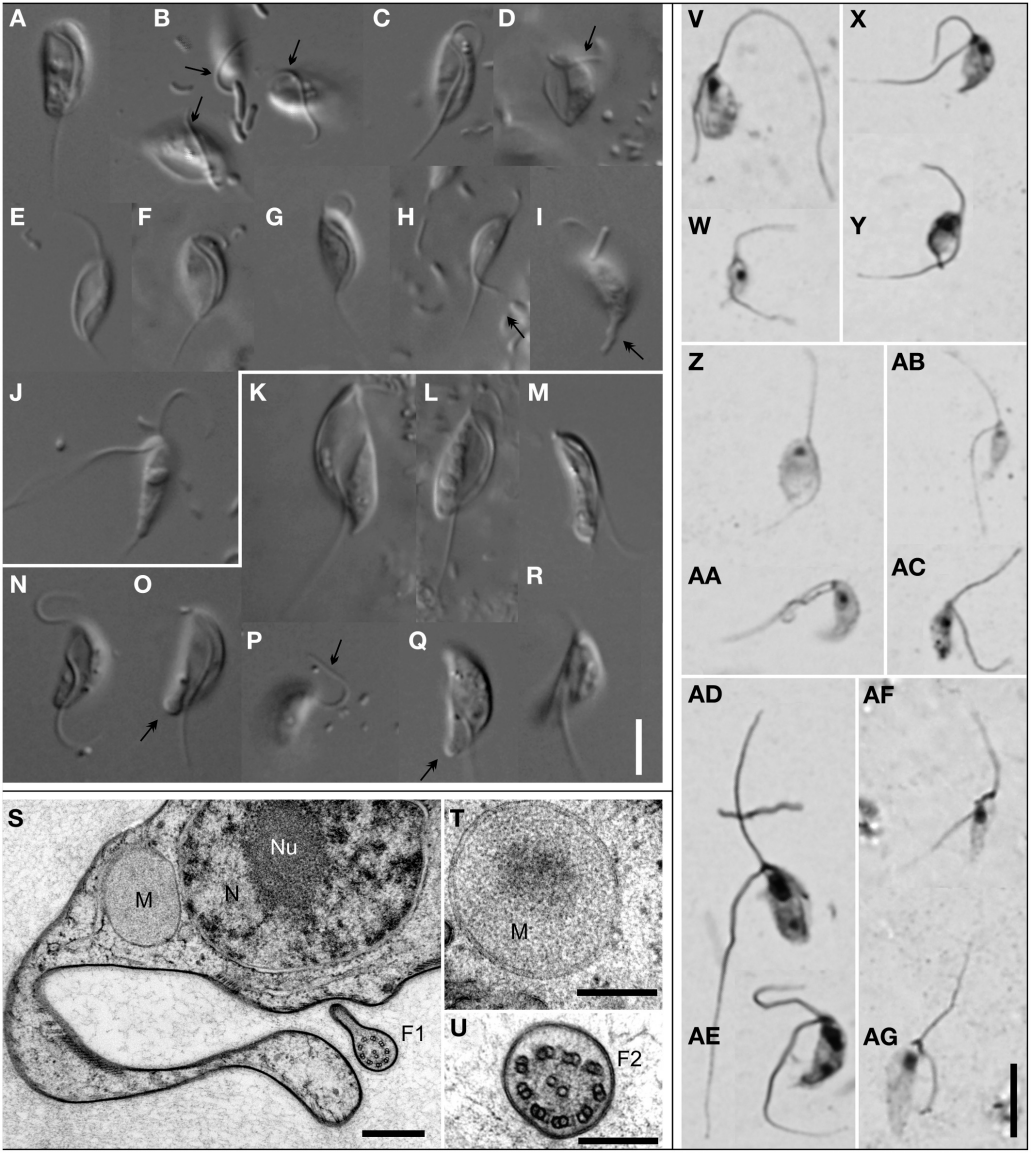
**Morphology of *Stygiellidae* fam. nov**. Living cells observed using Differential Interference Contrast **(A–R)**; protargol-stained cells observed by bright-field microscopy **(V–AG)**; TEM micrographs **(S–U)**. Most cells presented here are grooved cells, only a few are swimming cells **(J,M,R)**. Species and strains are arranged as follows: *Stygiella adhaerens* sp. nov. strain COORONG2 **(A,B)** and PETROCHORI **(Z,AA)**; *Stygiella incarcerata* comb. nov. strain NORMAR **(C,E,W)**, GOUVIA **(D)**, and EVROS1I **(V)**; *Stygiella cryptica* sp. nov. strain PC1 **(F,X,Y)**; *Stygiella agilis* sp. nov. strain AND **(G,J,AC)**, IGO3 **(H,I)**, and MANG **(AB)**; *Velundella trypanoides* gen. et sp. nov strain LUC3N **(K,L,S–U,AD,AE)** and BUSSPRAND **(M)**; *Velundella nauta* sp. nov. strain BMAND **(N–R,AF,AG)**. Bar = 5 μm **(A–R,V–AG)**, 500 nm **(S)**, and 200 nm **(T,U)**. Labels – F1, axoneme of the posterior flagellum possessing single dorsal vane; F2, axoneme of the anterior flagellum; M, mitochondrion-related organelle without cristae; N, nucleus; Nu, nucleolus; arrow, anterior flagellum attached to the substrate; double-arrow, cytoplasmic projections or pseudopodia on a cell posterior of Stygiellidae or a bulbous protrusion on cell posterior of *V. nauta* sp. nov.

The genus *Stygiella* (Figures 3A–J,V–AC) included 17 strains, 15 of which were isolated during this study. The strains were morphologically similar to each other, usually 6–9 μm long, crescent-shaped in lateral view, and possessing a broadly open, diamond-shaped groove that occupied either the entire or almost the entire ventral side of the grooved cell, reaching the posterior end. In the SSU rDNA tree, the genus *Stygiella* split into four lineages representing separate species. *S. incarcerata* (6.7–9.5 μm long) consisted of strains with relatively rare swimming cells and grooved cells that usually swam freely.

*Stygiella adhaerens* sp. nov. (5.0–8.4 μm long) and *S. cryptica* (6.4–10.5 μm) were morphologically very similar to each other and to *S. incarcerata*. Unlike most *S. incarcerata* strains, the grooved cells of both species very often adhered to the substrate by the anterior or the posterior flagellum and swam rarely. *S. cryptica* was the only stygiellid that possessed helix E23/3 in the hypervariable region V4 of SSU rRNA (see Supplementary Material S2, Figure S2.4).

In contrast to other *Stygiella* species, grooved cells of *S. agilis* sp. nov. (5.6–8.9 μm long) adhered to the substrate using the cell body (laterally or dorsally) and were somewhat narrower. Grooved cells swam rarely, while swimming cells were extremely abundant and constituted the dominant cell morphotype.

### *Velundella* gen. nov.

#### For extended version, see Supplementary Material S2, chapter 1.2.

The genus *Velundella* (Figures 3K–R,AD–AG) consists of two species: *V. nauta* and *V. trypanoides*; and environmental sequences closely related to the latter species. Generally, cells of *Velundella* spp. were conspicuously longer than those of *Stygiella*, usually 9–12 μm. Grooved cells possessed a distinct, spiral groove that did not reach the posterior end of the cell.

*V. trypanoides* cells (7.8–14.9 μm long) displayed a markedly spiral groove that almost reached the posterior end of the cell. The cells were noticeably elongated when compared with *Stygiella* spp. Grooved cells were broad and possessed a conspicuous groove, while the groove of serpentine-shaped swimming cells was less apparent and narrower. Virtually all grooved cells of *V. trypanoides* were attached to the substrate by the cell body or, sometimes, by posterior cytoplasmic projections.

*Velundella nauta* (8.4–11.8 μm long) possessed a less spiral and somewhat shorter groove than *V. trypanoides*. The majority of grooved cells were attached to the substrate by flagella, predominantly by the anterior one. The swimming cells possessed a distinctly shortened, narrow groove and sometimes also a bulbous protrusion at the posterior pole of the cell. Unlike the grooved cells, where the posterior flagellum of swimming cells exited the cell at its posterior pole, the posterior flagellum of the swimming cells left the shortened groove at a position ~½the cell length.

Transmission electron microscopy of *Velundella trypanoides* strain LUC3N (Figures 3S–U) showed that mitochondrion-related organelles lacked cristae and lay close to the nucleus. The single nucleus with central nucleolus contained peripheral heterochromatin, and the posterior flagellum bore a single vane on its dorsal side (up to at least 550 nm in diameter at its broadest).

### Pyrotags and their species affiliation

**Figure 4:**
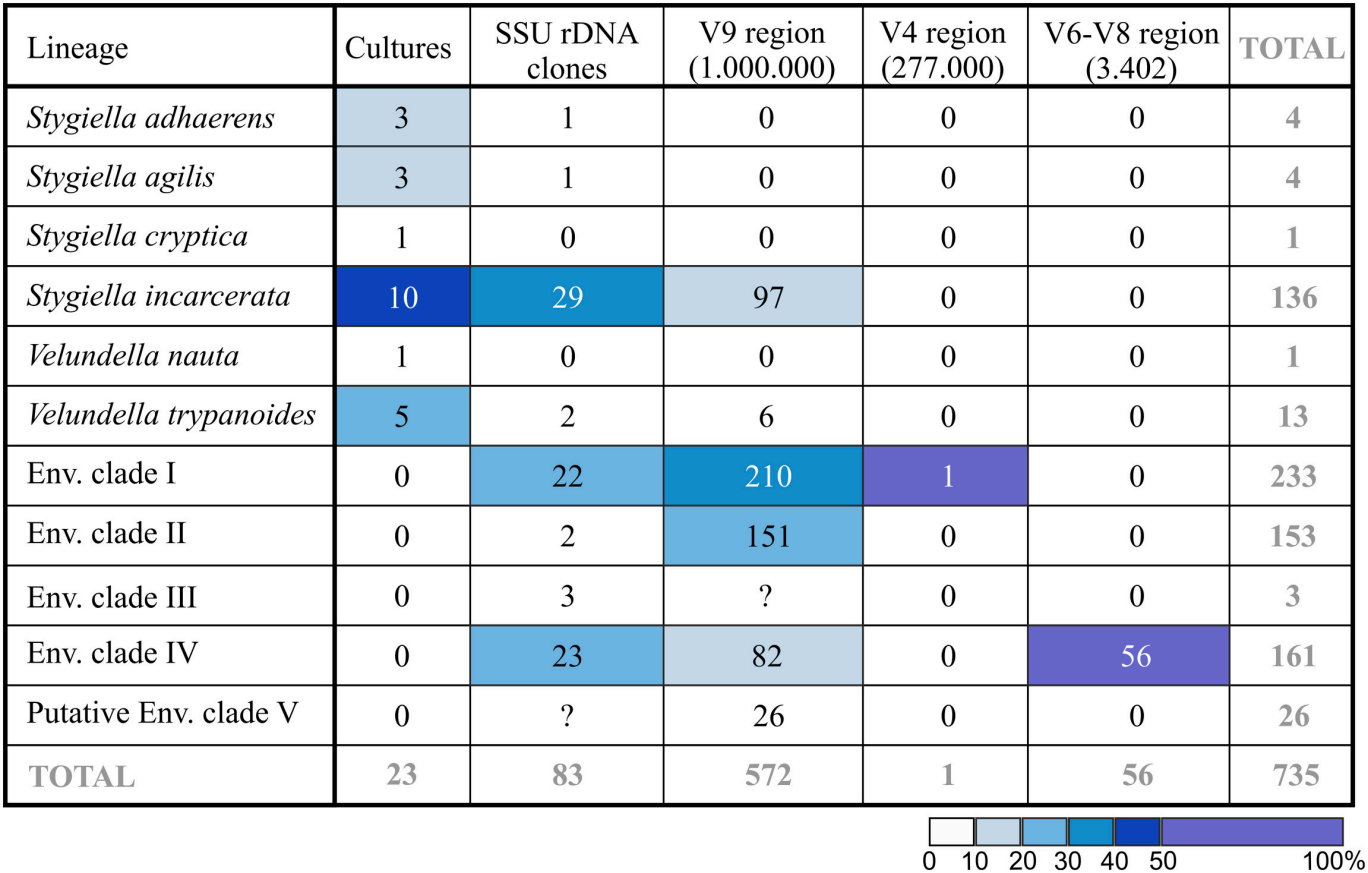
**Number of revealed sequences in each species/EC depending on method used**. Total number of protistan (V9 and V4 region of SSU rDNA) or eukaryotic (V6–V8 region of SSU rDNA) pyrotags is indicated in parentheses. Cell color indicates relative abundance (%) of the lineage in each column.

Aside from 83 environmental SSU rDNA clones obtained from GenBank (see above), we identified 629 stygiellid sequences (25 OTU_97_; see Supplementary Material S2, chapter 1.4.) after exhaustive data mining from anoxic/microoxic marine pyrotag archives from 145 samples from Cariaco Basin, Framvaren Fjord, Saanich Inlet, and three DHABs: Urania, Discovery and L'Atalante (Bernhard et al., 2014; Hallam et al., unpublished data deposited in GenBank as SAMN03387723, SAMN03387716, SAMN03387706, SAMN03387707, SAMN03387686, SAMN03387687, SAMN03387689, SAMN03387673, SAMN03387661, SAMN03387644, and SAMN03387630; Stoeck et al., 2009, 2010; Edgcomb et al., 2011b). These libraries together contained ~1,000,000 protistan sequences of the SSU rDNA hypervariable region V9, ~277,000 protistan sequences of the V4 region, and ~2,385,000 partial SSU rDNA sequences of V6–V8 regions of all three domains of life including 3402 eukaryotic reads. The total number of sequences in each species/environmental clade (EC) recovered with each method is presented in Figure 4.

The only sequence of Stygiellidae found among V4 sequences was 273 bp long and differed by three substitutions from sequences EF526735, DQ310256, and EF526837 (all of them represent EC I). Pyrotags of the V9 region were not longer than 210 bp, and their taxonomic affiliation was difficult to determine. Initially, we performed phylogenetic analysis of the V9 region including all available stygiellid sequences (see Supplementary Material S2, Figure S2.6). Genus *Stygiella* as well as EC II and IV were highly supported, while statistical support for other species was low. Therefore, we were able to determine species affiliation of only 233 pyrotags belonging to EC II or IV (five OTU_97_) and 97 pyrotags of *Stygiella* spp. (four OTU_97_). The species affiliation of other V9 pyrotags representing 14 different OTU_97_ remained unresolved. Conversely, we were able to assess species affiliation of almost all pyrotags by comparison of genetic distances among and within stygiellid species/environmental clades. Genetic distances between V9 pyrotags and sequences of particular species were lower or only slightly higher than intraspecific distances between sequences with clear species affiliations (see Supplementary Material S7). Only one group of four OTU_97_(26 pyrotags) was not assigned to a particular species/EC using this approach. This group is referred here to as EC V. The V6-V8 regions read library contained 56 jakobid reads (ca 480 bp in length). All of them represented a single genotype that belongs to EC IV and differed in a single nucleotide from the sequence EF526978.

### Distribution and salinity tolerance of stygiellidae

**Figure 5:**
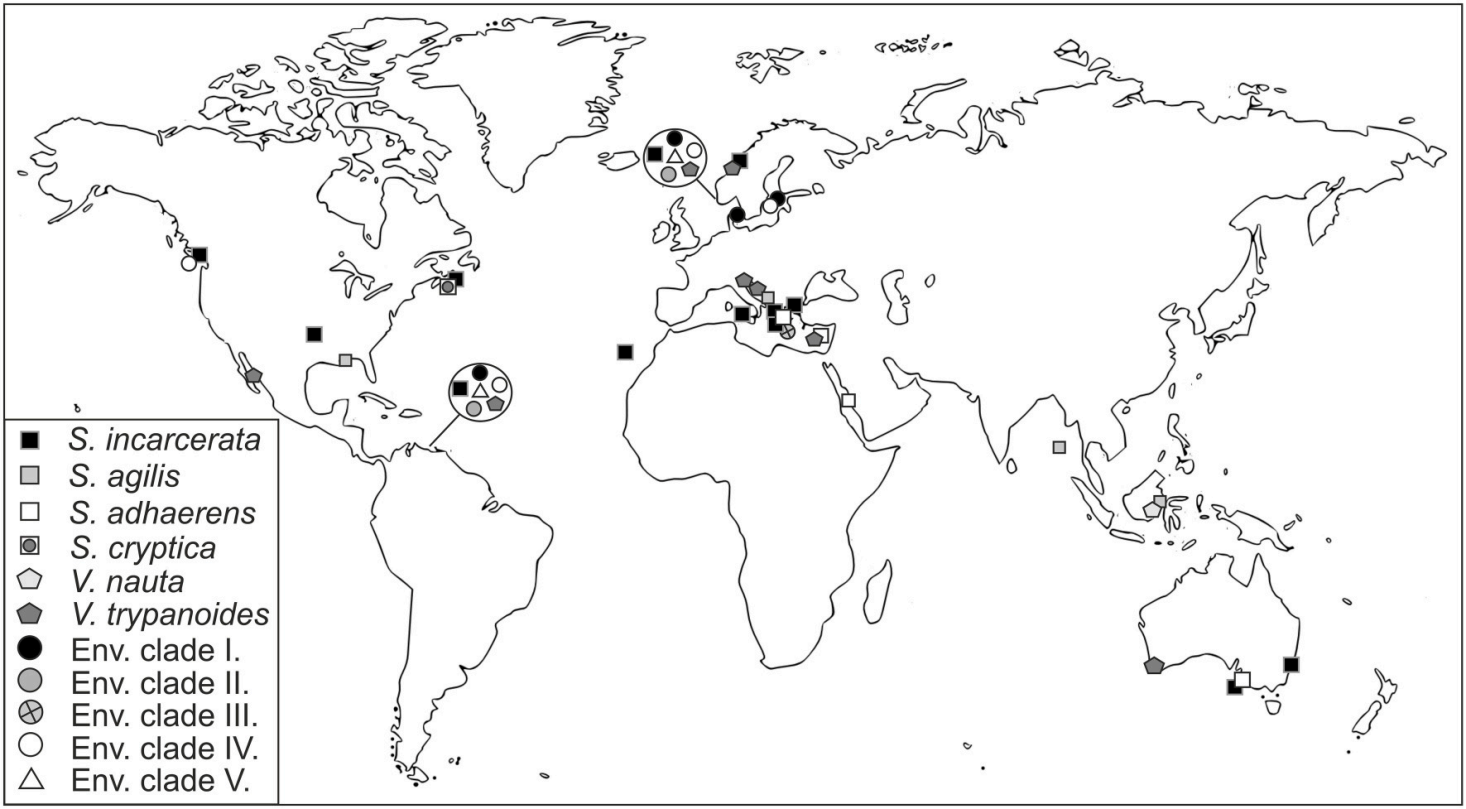
**Geographical distribution of the family Stygiellidae as revealed by environmental, PCR-based approaches and culture-based methods**.

Stygiellidae have been found in marine, oxygen-poor habitats, often in the presence of sulfides, ammonium or methane (see Supplementary Material S6). However, sequences belonging to EC IV have also been reported from oxic waters of Saanich Inlet (c0_2_ > 100 μM) with non-detectable amounts of sulfide and ammonium (see Supplementary Material S6 for details). All natural localities where stygiellids were detected by traditional or environmental approaches, are summarized in Figure 5.

**Figure 6:**
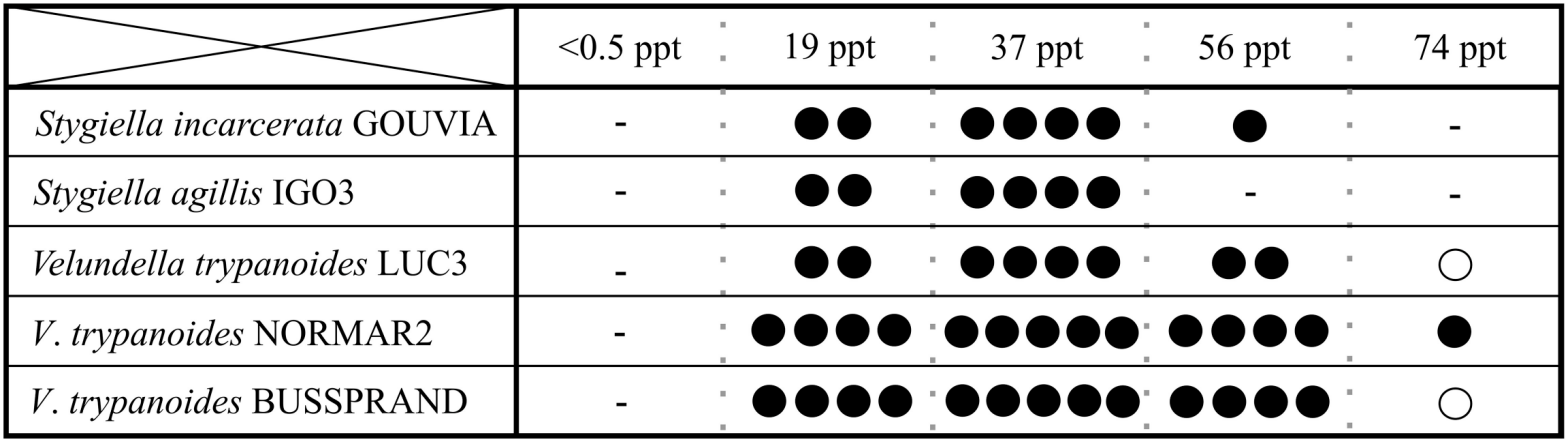
**Salinity ranges for growth of three Stygiellidae species (*Stygiella incarcerata, S*. *agilis*, and *Velundella trypanoides*)**. Number of black dots indicates relative cell density in the culture (the higher number, the higher density); a circle indicate presence in very low densities accompanied by inability of active growth.

To reveal differences in salinity tolerance among and within species of Stygiellidae, as well as their ability to survive in freshwater habitats, we examined five monoeukaryotic cultures of *Stygiella incarcerata, S. agilis*, and *Velundella trypanoides*. None was able to grow in salinity corresponding to fresh water. We also detected differences in salinity tolerance among examined species (see Figure 6).

## Discussion

### Phylogenetic implications

Our phylogenetic analyses indicate that all jakobids detected in anoxic habitats constitute a clade, the Stygiellidae, that represents a sister lineage to the Andaluciidae, aerobic protists reported from soils and alkaline lakes (Figures 1, 2). Together, Stygiellidae and Andaluciidae form Andalucina, one of the two jakobid lineages (Burger et al., 2013; Cavalier-Smith, 2013; this study). Monophyly of Andalucina is clearly validated by several lines of evidence: (1) the SSU rDNA phylogeny (Figure 1), (2) the phylogenetic analysis based on six protein-coding genes (Figure 2), (3) the α-tubulin gene phylogeny (Supplementary Material S2, Figure S2.7.), and (4) the unique C:G base pair within the basal stem of helix 27 in the SSU rRNA molecule as defined by Lara et al. (2006).

Our strains span two of the 6–7 clades that we identified within the Stygiellidae. These two cultured clades are described here as *Velundella* and *Stygiella*. The other stygiellids still remain uncultured (EC I–V), and their relationship to *Velundella* and *Stygiella* is unresolved. EC II–IV form a monophyletic group, but their close relationship to EC I was not sufficiently supported. The phylogenetic position of EC V is even more uncertain, because only a very short portion of its SSU rDNA sequence is available (172 bp). Moreover, it was not possible to compare EC V to EC III because their SSU rDNA sequences do not overlap.

### Molecular and species diversity of stygiellidae

In order to reveal species diversity within Stygiellidae, we determined the species boundaries among our strains using phenotypic characterization in combination with SSU rDNA analysis. This approach indicates that our isolates represent six separate species. As a next step, we assessed species identity of available SSU rDNA sequences obtained by environmental PCR-based approaches. For deep-branching environmental lineages, we established provisional, monophyletic groups referred to here as EC I–IV. Intraspecific genetic distances among SSU rDNA of stygiellids did not exceed 4.6%, and the minimum interspecific distance was 6.7%. Minimum SSU rDNA distances between EC I–IV were greater than 6.8%. This was significantly greater than that observed for intraspecific distances, and is comparable to the distance between particular *Velundella* or *Stygiella* species. We therefore assumed that the four environmental clades represent separate species, and Stygiellidae comprises at least 10 species in total. This means that Stygiellidae contains at least the same number of species that has previously been described for all of Jakobida. Stygiellid species are morphologically very similar, but genetically and ecologically diverse. Our data further indicate that particular species of Stygiellidae also differ in salinity tolerance or tendency and style of their attachment to the substrate.

### Ecological diversity of stygiellidae

Stygiellid sequences were found almost exclusively in environmental libraries from anoxic or microoxic sites (see results for references). DNA-based data from Saanich Inlet represent the only exception (Hallam et al., unpublished data), because a single OTU of Stygiellidae was detected not only in anoxic or microoxic samples, but also in oxic waters. This observation could be explained in several ways, e.g., if this taxon is either allochtonous to oxic waters in Saanich, particle associated, and therefore, protected from oxygen exposure, or is delivered into oxic waters due to seasonal turnovers that occur in Saanich Inlet. One of these explanations seems likely because this OTU belongs to the EC IV that has been repeatedly detected in anoxic, sulfide-rich environments in DNA- and RNA-based studies. RNA molecules are thought to better target metabolically active, indigenous members of microbial communities compared to DNA (Stoeck et al., 2007). However, Stygiellid ribosomal RNA has been reported from habitats with oxygen concentrations up to 19.8 μmol/l. Experimental data from *Stygiella* and *Velundella* also support the anaerobic lifestyle of stygiellids since their mitochondria are acristate, and the cells are not able to grow under fully-oxic conditions (Simpson and Patterson, 2001; this study).

We detected active cells of Stygiellidae in a wide range of marine environments including brackish or hypersaline waters and inland salt springs, but they most likely do not inhabit freshwater sites. This assumption is supported by three independent lines of evidence: (1) their sequences are absent from freshwater clone libraries, (2) isolation of Stygiellidae from freshwater anoxic habitats was unsuccessful, and (3) our strains of Stygiellidae were unable to grow in the freshwater medium. Three examined species of the genera *Velundella* and *Stygiella* thrive in salinities ranging from 19 to 74 ppt, or 19 and 56 ppt, respectively. While the observed ecophysiological variability of strains we examined may be influenced by the presence of different species of bacterial prey in their respective cultures (various undefined species), the same level of salinity tolerance of three *V. trypanoides* strains examined suggests that the impact of bacterial prey is probably not the main driving factor.

RNA-based environmental data further suggest an extraordinary tolerance of some stygiellids (EC III) to a wide range of environmental conditions (see Supplementary Material S6), although it has to be validated experimentally. Stygiellidae flourish in sulfidic environments and constitute an important component of eukaryotic communities in anoxic and sulfidic marine habitats. This is shown by both DNA- and RNA-based environmental studies of the Gotland Deep (Stock et al., 2009; Weber et al., 2014). In terms of clone abundance, stygiellids constituted a major component of eukaryotic communities in anoxic, sulfidic layers of Gotland Deep, where samples dominated by stygiellids were collected from a number of different sampling sites in two different years. Additionally, stygiellids were frequently detected in marine anoxic habitats using not only molecular, but also culture-based approaches (see Results and Supplementary Material S6).

Sulfide-rich, anoxic Framvaren Fjord (Norway) and Cariaco Basin (Venezuela) provide ideal model systems for studying the biodiversity of anaerobic protists including stygiellids; both localities are permanently anoxic, sulfide-rich, and extensive environmental data from a number of sampling sites at each location have been published. When we searched available data sets from those two locations for stygiellids, we detected the same six species in both geographically distant, but ecologically similar habitats (Figure 5). In addition, we showed that different stygiellids often co-occur in a single microhabitat. A striking example of this is the F2 sampling site in Framvaren Fjord (Stoeck et al., 2009), where DNA sequences of five different stygiellid species were detected. Our culture-based data further support the co-occurrence of different stygiellids, since different species or genera were cultured from a single sample, ca 2 ml of sediment (NORMAR/NORMAR2; COORONG/COORONG2), and the original culture of EVROS1 contained two different phylotypes of *Stygiella incarcerata* (EVROS1I, EVROS1T).

### Culture-based vs. culture-independent methods of diversity detection

Clone and pyrotag DNA-based libraries from anoxic marine habitats are typically dominated by alveolates, stramenopiles, and rhizarians (Stoeck et al., 2010; Edgcomb et al., 2011b; Orsi et al., 2012) and contain relatively small portions of sequences affiliated to Excavata. In fact, environmental studies do not significantly contribute to unveiling species diversity of the excavates, except for euglenozoans and jakobids (Orsi et al., 2011; this study). While this may be due in some unknown extent to primer biases against this group, culture-based approaches consistently reveal novel species or even deep-branching lineages of obligatorily anaerobic marine excavates (Kolisko et al., 2010; Yubuki et al., 2010; Pánek et al., 2014a,b).

To reveal the full diversity of species of anaerobic jakobids and to compare the efficiency of culture-based and culture-independent techniques, we analyzed sequences obtained by three methodologically different approaches: (1) cell culturing and subsequent DNA sequencing; (2) direct extraction of nucleic acid from the environment, amplification and clone sequencing; and (3) next generation sequencing (NGS) of amplified DNA obtained from the environment.

Results of both molecular-based environmental approaches correspond to each other regarding the most abundant jakobid species in nature: EC I, IV, and *Stygiella incarcerata*. NGS methods also detect a high abundance of EC II. From those, only *S. incarcerata* is represented in our culture collection and constitutes 43% of the strains (the most abundant cultured species). Interestingly, the other five cultured species represent together only 1.4% of stygiellid environmental sequences (Figure 4).

Culture-based approaches have been able to span the entire species diversity of two identified Stygiellidae clades (*Stygiella* and *Velundella*) including two species that have never been detected by culture-independent approaches: *V. nauta* and *S. cryptica*. Short read archives do not contain any *Stygiella* and *Velundella* species aside from *S. incarcerata* and *V. trypanoides*. In contrast, culture-based approaches were unable to detect any of Stygiellidae environmental clades revealed by other methods (EC I-V). Discrepancies between molecular- and culture-based results are likely at least in part attributable to biases of PCR primers as well as biases of selective culture media and conditions. Generally, we can interpret discrepancies between culture-based and environmental approaches in three different ways: (1) If taxa are present at very low abundances in otherwise diverse environmental samples, it is possible that sequencing depth will not be great enough to detect them. In other words, they may be common in anoxic localities worldwide in terms of presence, but rare in terms of local abundance. Culture-based approaches may therefore be more efficient for detection of rare species, especially if low abundance of the species is combined with primer or sampling method biases. We consider such a scenario plausible since it is quite easy to establish cultures of certain protistan species even from a single cell (e.g., Hess et al., 2006). (2) These species are globally distributed, but extremely rare or even missing from many anoxic habitats, but locally abundant under specific conditions. Culture-based methods would detect such species more frequently, as more localities with conditions suitable for growth of the species were explored. (3) These species are quite common, their absence in environmental studies is an artifact of primer or sampling method biases. However, sampling method bias is less plausible in the case of *S. agilis*, which only rarely attaches to the substrate and should be easily detectable in the sample. Sequences of all cultured species are compatible at least with some commonly used primer sets in environmental, PCR-based studies (e.g., (Behnke et al., 2006; Edgcomb et al., 2011b; Orsi et al., 2012)), but nothing is known about the number of rDNA copies in their genomes.

Despite the inability of environmental, PCR-based approaches to discover the full diversity of stygiellids, we showed that these methods are relatively powerful for revealing the full species diversity of the anaerobic jakobids, since they detected ~80% of the known species diversity of the group. Thus, Stygiellidae seems to be a suitable model group for studying the biogeography and role of bacterivorous nanoflagellates in marine anoxic communities using these approaches. Interestingly, culture-based approaches were more successful in revealing species diversity of two particular subclades, genera *Stygiella* and *Velundella*.

Our data suggests that anaerobic jakobids are much more diverse than previously expected, although their overall diversity is relatively low (tens rather than hundreds of species). Some stygiellid species or environmental clades are very common in nature (e.g., *Stygiella incarcerata*). By contrast, *Velundella nauta* or *Stygiella cryptica* appears to belong to the “rare biosphere.”

The ratio of diversity of anaerobic jakobids detected in environmental sequence libraries to the total number of detected jakobid species is inconsistent with data from CLOs (free-living relatives of diplomonads), where environmental studies have been unsuccessful in revealing most of the diversity described by culture-based methods (Kolisko et al., 2010). This likely reflects biases of the most common PCR primers used in those surveys against CLOs. It is therefore necessary to combine environmental data with data from classical, culture-based approaches to obtain the fullest representation of species, particularly when examining taxonomic groups that may be largely missed by current PCR-based approaches.

### Taxonomic summary

*Type material consists of protargol preparations deposited in the collection of the Department of Parasitology, Charles University in Prague, Czech Republic. Catalog numbers are given for each species. For additional information see extended version of the taxonomic summary in the Supplementary Material S2, chapter 1.5*.

***Stygiellidae* fam. nov**. Description: Aloricate marine jakobids with acristate mitochondria. Type genus: *Stygiella* gen. nov. Zoobank registration: urn:lsid:zoobank.org:act:4A238129-B037-4B4E-9D15-E205E7B13605. ***Stygiella* gen. nov**. Description: see results. Type species: *Jakoba incarcerata* Bernard Simpson & Patterson, 2000 (= *Stygiella incarcerata* comb. nov.). Zoobank registration: urn:lsid:zoobank.org:act:9EA9ADF7-11C7-40D7-925F-7845D48420C0. ***Stygiella adhaerens* sp. nov**. Description: see results. Syntype: 12/96 (PETROCHORI). Zoobank registration: urn:lsid: zoobank.org:act:E2EF3439-3683-42F1-8985-5A550683F374. ***Stygiella agilis* sp. nov**. Description: see results. Syntype: 9/30-32 and 10/99, 100 (AND). Zoobank registration: urn:lsid:zoobank.org:act:A0114EE7-55D7-4960-8B92-E1AC20019774. ***Stygiella cryptica* sp. nov**. Description: see results. Syntype: 5/97 and 6/51, 52 (PC1).Zoobank registration: urn:lsid:zoobank.org: act:21C5B523-3A4E-405C-A7B4-6507E31F3C1B. ***Velundella* gen. nov**. Description: see results. Type species: *Velundella trypanoides* sp. nov. Zoobank registration: urn:lsid:zoobank.org:act:9472415A-259E-484C-83B2-E0D3B273CD32. ***Velundella trypanoides* sp. nov**. Description: see results. Syntype: 6/27, 28, 84, 85 (LUC3N). Zoobank registration: urn:lsid:zoobank.org:act:3879E18C-D7F9-4D48-B0B4-EEF635D39664. ***Velundella nauta* sp. nov**. Description: see results. Syntype: 9/92, 93 (BMAND). Zoobank registration: urn:lsid:zoobank.org:act:3879E18C-D7F9-4D48-B0B4-EEF635D39664.

## Author contributions

TP and IČ designed research; PT, TP, and MH performed research; TP, MP, IČ, VE, and ČV analyzed data and contributed to the paper.

### Conflict of interest statement

The authors declare that the research was conducted in the absence of any commercial or financial relationships that could be construed as a potential conflict of interest.
