# Supplementary material for: Combined Culture-Based and Culture-Independent Approaches Provide Insights into Diversity of Jakobids, an Extremely Plesiomorphic Eukaryotic Lineage
Source: Front Microbiol. 2015 Nov 18;6:1288. doi: 10.3389/fmicb.2015.01288 (PMC4649034; doi:10.3389/fmicb.2015.01288)
Supplement: Supplementary file 1 [file DataSheet1.DOCX]

Supplementary Material S1 - Methods

**Combined culture-based and culture-independent approaches provide insights into diversity of jakobids, extremely plesiomorphic eukaryotic lineage**

Tomáš Pánek^*^, Petr Táborský, Maria G. Pachiadaki, Miluše Hroudová, Čestmír Vlček, Virginia P. Edgcomb & Ivan Čepička

***Correspondence:** Tomáš Pánek, [mistrpanek@seznam.cz](mailto:mistrpanek@seznam.cz)

CONTENTS

[1 Supplementary text 1](#_Toc426726769)

[1.1 List of strains included in the study 1](#_Toc426726770)

[1.2 Culture media used in this study 2](#_Toc426726771)

[1.2.1 ATCC 802 freshwater medium (modified) 2](#_Toc426726772)

[1.2.2 ATCC 1525 seawater medium (modified) 2](#_Toc426726773)

[1.2.3. 2X Modified ATCC 1525 medium 2](#_Toc426726774)

[1.2.4. Cerophyll-based media with salinity range from fresh water to brine water 3](#_Toc426726775)

[1.3 Nucleic acid extraction, PCR amplification, Sanger and 454 sequencing 3](#_Toc426726776)

[1.4 SSU rDNA dataset construction 3](#_Toc426726777)

[1.5 Protein datasets construction 4](#_Toc426726778)

[1.6 Primers used in this study 4](#_Toc426726779)

[2 Supplementary tables 5](#_Toc426726780)

[3 Supplementary references 6](#_Toc426726781)

# Supplementary text

## List of strains included in the study

Strains were isolated between 2008 and 2012 and were maintained in polyxenic agnothobiotic cultures with unidentified bacteria at room temperature. They were subcultured once a week. Most cultures were not monoeukaryotic and contained various other protists besides the jakobids (see the Supplementary Table S1.1 below). Strains EVROS1T and EVROS1I (both *S*. *incarcerata* comb. nov.) were successfully separated and cultured individually after several tens of initial passages. Strains COORONG (*S*. *incarcerata* comb. nov.) and COORONG2 (*S*. *adhaerens* sp. nov.) were isolated from a single sample that was inoculated into two tubes containing ATCC medium 1525, and two separate cultures were created. Strains NORMAR (*S*. *incarcerata* comb. nov.) and NORMAR2 (*V*. *trypanoides* n. gen., n. sp.) were isolated from the same culture.

## Culture media used in this study

## 1.2.1 ATCC 802 freshwater medium (modified)

Add cerophyll (2.5 g) to 1000 ml of distilled water and boil for 5 minutes. Filter through Whatman #1 filter paper. Add 0.5 g Na_2_HPO_4_ and distilled water to compensate evaporation. *Autoclave for 15 – 20 minutes at 121^o^C. Store at 4^o^C.*

## 1.2.2 ATCC 1525 seawater medium (modified)

Solution 1:

Add cerophyll (2.5 g) to 500 ml of distilled water and boil for 5 minutes. Filter through Whatman #1 filter paper. Add distilled water to compensate evaporation. Autoclave for 15 – 20 minutes at 121^o^C.

Solution 2:

Prepare 300 ml of solution containing NaCl (24.72 g), KCl (0.68 g), CaCl_2_.2H_2_O (1.36 g), and MgCl_2_.6H_2_O (4.66 g). Autoclave for 15 – 20 minutes at 121^o^C.

Solution 3:

Prepare 100 ml of solution containing MgSO_4_.7H_2_O (6.29 g). Autoclave for 15 – 20 minutes at 121^o^C.

Solution 4:

Prepare 100 ml of solution containing NaHCO_3_ (0.18 g). Autoclave for 15 – 20 minutes at 121^o^C.

*When cool, aseptically combine all four solutions. Chlorides, NaHCO_3_ and sulfide is autoclaved separately to avoid salt precipitation. Store at 4^o^C.*

## 1.2.3. 2X Modified ATCC 1525 medium

Solution 1:

Add cerophyll (2.5 g) to 500 ml of distilled water and boil for 5 minutes. Filter through Whatman #1 filter paper. Add distilled water to compensate evaporation. Autoclave for 15 – 20 minutes at 121^o^C.

Solution 2:

Prepare 300 ml of solution containing NaCl (49.44 g), KCl (1.36 g), CaCl_2_.2H_2_O (2.72 g), and MgCl_2_.6H_2_O (9.32 g). Autoclave for 15 – 20 minutes at 121^o^C.

Solution 3:

Prepare 100 ml of solution containing MgSO_4_.7H_2_O (12.58 g). Autoclave for 15 minutes at 121^o^C.

Solution 4:

Prepare 100 ml of solution containing NaHCO_3_ (0.36 g). Autoclave for 15 – 20 minutes at 121^o^C.

*When cool, aseptically combine all four solutions. Chlorides, NaHCO_3_ and sulfide is autoclaved separately to avoid salt precipitation. Store at 4^o^C.*

## 1.2.4. Cerophyll-based media with salinity range from fresh water to brine water

1. Freshwater: prepared as ATCC 802 freshwater medium (modified)
2. Brackish (19ppt) medium: prepared as 1:1 combination of ATCC 802 freshwater medium and ATCC 1525 seawater medium
3. Normal seawater (37ppt) medium: prepared as ATCC 1525 seawater medium
4. Brine (56ppt) medium: prepared as 1:1 combination of ATCC 1525 seawater medium and 2X ATCC 1525 seawater medium
5. Brine (74ppt) medium: prepared as 2X ATCC 1525 seawater medium

## Nucleic acid extraction, PCR amplification, Sanger and 454 sequencing

**Genomic DNA** was isolated using the ZR Genomic DNA II Kit™ (Zymo Research, USA). Almost complete SSU rRNA gene was amplified using universal eukaryotic primers A and B (Medlin *et* *al*., 1988). The alpha-tubulin gene of *Velundella* *trypanoides* sp. nov. LUC3N and *Stygiella* *cryptica* sp. nov. PC1 was amplified using nested PCR and two different sets of primers (Edgcomb *et* *al*., 2001; Yoon *et* *al*., 2008); for primer sequences see below. The PCR products were purified and directly sequenced or cloned into the pGEM®-T EASY vector (Promega); at least two clones were sequenced.

**Total RNA** was extracted from cultured cells of *Velundella trypanoides* LUC3N (monoeukaryotic polyxenic culture) using the NucleoSpin RNA II kit (Macherey-Nagel). mRNA was selected with Dynabeads Oligo(dT)_25_ (Invitrogen) and used for cDNA construction by SMARTer PCR cDNA Synthesis Kit (Clontech) following the manufacturer's instructions with the exception of using modified CDS-T22 primer (5'-AAGCAGTGGTATCAACGCAGAGTTTTTGTTTTTTTCTTTTTT TTTTVN-3') instead of 3' BD SMART CDS Primer II A, first strand synthesis time was prolonged to 2 hours, and PCR conditions during second strand synthesis were modified (denaturation: 95°C/ 2 min, amplification: 24 times 95°C/ 10 sec, 65°C/ 30 sec, 72°C/ 3 min, final extention: 72°C/ 5 min). cDNA was normalized by Trimmer cDNA Normalization Kit (Evrogen). Normalized cDNA was used for preparation of sequencing library by Rapid Library Preparation Method (Roche) and sequenced using GS FLX+ chemistry followed by standard analysis of signal processing (454 Life Sciences, Roche). Automatic assembly was performed by Newbler 2.6 software.

ESTs of *Velundella* *trypanoides* gen. nov. sp. nov. (strain LUC3N) were screened for previously published *Stygiella* *incarcerata* comb. nov. gene orthologues of α-tubulin, β-tubulin, cytosolic HSP70, cytosolic HSP90, EF-2, and EF-1α genes using local BLAST (tBLASTN) in BioEdit 7.0.4.1. The tBLASTN hits were then translated to amino acid residues, using ExPASy SIB Bioinformatics Resource Portal (web.expasy.org/translate/).

## SSU rDNA dataset construction

Exhaustive BLASTn searches (Altschul *et al.,* 1990) were performed using new and existing Andalucina SSU rDNA sequences to identify all clone sequences from environmental libraries present in the GenBank database (see supplementary material S3). The presence of sequence chimeras was assessed by visual screening of the sequence alignment and subsequent BLASTn searching using contradictory parts of any problematic sequences. This approach identified environmental sequences AY046663, AY916611, AY916612, and AY916613 as chimeras. From chimeric sequences that provided conflicting taxonomic signal (BLASTn searching), we included only the part that was clearly recognized as a part of jakobid SSU rRNA gene.

Preliminary phylogenetic analysis was based on the dataset that contained 83 environmental jakobid clones from anoxic and microoxic samples and several jakobid sequences as outgroups (phylogenetic tree computed in RAxML is deposited in the supplementary material S4). To the final dataset, we included 123 OTUs with sequences longer than 490 bp: 95 jakobid sequences from GenBank, sequences of 21 new strains of Stygiellidae fam. nov., and 6 eukaryotic sequences from GenBank as outgroup. The final dataset contained 1588 aligned nucleotide positions (**supplementary material S1A**, see below).

## Protein datasets construction

Inferred Amino acid sequences obtained from *Velundella* *trypanoides* LUC3N were added to single-gene protein datasets containing sequences adopted from seed datasets published by Brown *et al.*, 2013 and Yabuki *et al.*, 2014, or sequences previously deposited in GenBank. To improve taxon sampling of our dataset, we further found genes of interest in ESTs of three different amoebozoans deposited in MMETSP (Keeling *et al.*, 2014): *Mayorella* sp. ATCC 50980, *Filamoeba* *nolandi* ATCC 50430, and *Pessonella* sp. PRA-29.

All amino acid datasets were subsequently aligned with the help of the MAFFT 7.110 server at default settings and tested for undetected paralogs or contaminants using phylogenetic analyses of the alignments. Single-gene trees were checked manually. A final multi-protein dataset contained 3200 aligned characters (amino acid residues) of six protein coding genes: actin, β-tubulin, cytosolic HSP70, cytosolic HSP90, EF-2, and EF-1α. The best partitioning scheme for multi-protein data set was computed in PartitionFinderProtein 1.1.0 (Lanfear *et* *al*., 2012) under the Bayesian Information Criterion and greedy searching. Final partitioning scheme was based on PROTGAMMAILG model and set as follows: LG, p1 = 1-360, 361-792; LG, p2 = 793-1224, 2013-2586; LGF, p3 = 1225-2012; LG, p4 = 2587-3200. Gene for α-tubulin was analysed separately (for reasons see results). The final multi-protein dataset containing 47 taxa is deposited as **supplementary material S1B** (see below), details on the source sequence data for each taxon is available **as supplementary material S1C**  (see below).

## Primers used in this study

Almost complete SSU rDNA was amplified from genomic DNA using primers A and B with an annealing temperature of 50 ^o^C. The alpha-tubulin gene of *Velundella* *trypanoides* LUC3N and *Stygiella* *cryptica* PC1 was amplified from genomic DNA using nested PCR with annealing temperature of 50 ^o^C in both PCRs. The primary PCR was carried out with primers ATUB_A and ATUB_B; 0.5 µl of the purified PCR product was used as the template for the secondary PCR using primers ATUB_secF and ATUB_secR. The beta-tubulin gene of *Velundella* *trypanoides* LUC3N was amplified from genomic DNA using primers BTUB_A and BTUB_B with annealing temperature of 56.8 ^o^C. List of used primers is presented below (Supplementary Table S1.2).

# Supplementary tables

**Supplementary Table S1.1. List of strains.** Monoeukaryotic strains are marked by asterisks. (^1^) The strain was derived from the same isolate as EVROS1I published by Pánek et al. (2014). (^2^) The strain was derived from the same isolate as GOUVIA published by Pánek et al. (2014). (^3^)The strain was derived from the same isolate as AND published by Pánek et al. (2014). (^4^)The strain was derived from the same isolate as LARNAKA2 published by Kolisko et al. (2010).

| **Strain** | **Locality** | **Coordinates** |
| --- | --- | --- |
| ***Stygiella incarcerata* (former *Andalucia incarcerata*)** | | |
| COORONG | Coorong NP, Australia | 36^o^04’S, 139^o^35’E |
| EVROS1I^1^ | Evros delta, Greece | 40^o^48’N, 26^o^01’E |
| EVROS1T | Evros delta, Greece | 40^o^48’N, 26^o^01’E |
| FUEN2 | Punta de Fuencaliente, La Palma, Canary Islands, Spain | 28^o^27’N, 17^o^50’W |
| GOUVIA^2^ | Corfu Island, Greece | 39^o^38’N, 19^o^50’E |
| NORMAR | Nørdre Eidsbukta, Aure, Norway | 63 ^o^18’N, 8 ^o^32’E |
| KALOGRIA^*^ | Kalogria, Greece | 38^o^09’N, 21^o^22’E |
| OROSEI | Orosei, Sardinia, Italy | 40^o^33’N, 9^o^68’E |
| ***Stygiella adhaerens sp. nov.*** | | |
| COORONG2 | Coorong NP, Australia | 36^o^04’S, 139^o^35’E |
| LARNAKA2N^4^ | Larnaka, Cyprus | 34^o^51’N, 33^o^37’E |
| PETROCHORI | Pilos, Petrochori, Greece | 36^o^58’N, 21^o^39’E |
| ***Stygiella cryptica sp. nov.*** | | |
| PC1^*^ | Peggy’s Cove, Canada | 44^o^29’N, 63^o^55’W |
| ***Stygiella agilis sp. nov.*** | | |
| AND^3^ | Elephant beach, Havelock Island, Andaman Islands, India | 12^o^00’N, 92^o^56’E |
| IGO3^2*^ | Igoumenitsa, Greece | 39^o^31’N, 20^o^14’E |
| MANG | Balikpapan Bay, Borneo, Indonesia | 1°03' S, 116°42' E |
| ***Velundella nauta* sp. nov.** | | |
| BMAND | Balikpapan, Kalimantan, Indonesia | 1°20' S, 116°50' E |
| ***Velundella trypanoides* sp. nov.** | | |
| BUSSPRAND^*^ | Marrybrook, Busselton, Australia | 33^o^38’S, 115^o^11’E |
| LARNAKA^*^ | Larnaka, Cyprus | 34^o^51’N, 33^o^37’E |
| LUC3N^*^ | Brač island, Croatia | 43^o^17’N, 16^o^52’E |
| MURANO3 | Murano Islands, Venetian lagoon, Italy | 45^o^27’N, 12^o^21’E |
| NORMAR2 | Nørdre Eidsbukta, Aure, Norway | 63 ^o^18’N, 8 ^o^32’E |

**Supplementary Table S1.2. List of primers used in the study.**

| **NAME** | **REFERENCE** | **PRIMER SEQUENCE (5´- 3´)** | **TARGET GENE** |
| --- | --- | --- | --- |
| A | Medlin *et* *al*., 1988 | AYCTGGTTGAYYTGCCAG (F) | SSU rDNA |
| B | Medlin *et* *al*., 1988 | TGATCCTTCTGCAGGTCCACCTAC (R) | SSU rDNA |
| ATUB_secF | Yoon *et* *al*., 2008 | TTGTACTGCYTNGARCAYG (F) | Alpha-tubulin gene |
| ATUB_secR | Yoon *et* *al*., 2008 | ACGTACCAGTGNACRAANGC (R) | Alpha-tubulin gene |
| ATUB_A | Edgcomb *et* *al*., 2001 | RGTNGGNAAYGCNTGYTGGGA (F) | Alpha-tubulin gene |
| ATUB_B | Edgcomb *et* *al*., 2001 | CCATNCCYTCNCCNACNTACCA (R) | Alpha-tubulin gene |
| BTUB_A | Edgcomb *et al*., 2001 | GCAGGNCARTGYGGNAAYCA (F) | Beta-tubulin gene |
| BTUB_B | Edgcomb *et al*., 2001 | AGTRAAYTCCATYTCRTCCAT (R) | Beta-tubulin gene |

# Supplementary references

Altschul SF, Gish W, Miller W, Myers EW, Lipman DJ. (1990). Basic local alignment search tool. J Mol Biol 215: 403–410.

Brown MW, Sharpe SC, Silberman JD, Heiss AA, Lang BF, Simpson AG, Roger AJ. (2013). Phylogenomics demonstrates that breviate flagellates are related to opisthokonts and apusomonads. Proc R Soc B 280: 20131755.

Keeling PJ, Burki F, Wilcox HM, Allam B, Allen EE, Amaral-Zettler LA, ... & Murray S. (2014). The marine microbial eukaryote transcriptome sequencing project (MMETSP): illuminating the functional diversity of eukaryotic life in the oceans through transcriptome sequencing. PLoS Biology 12: e1001889.

Kolisko M, Silberman JD, Cepicka, I, Yubuki N, Takishita K, Yabuki A, Leander BS, Inouye I, Inagaki Y, Roger AJ, Simpson AGB. (2010). A wide diversity of previously undetected free-living relatives of diplomonads isolated from marine/saline habitats. Environ Microbiol 12: 2700–2710.

Lanfear R, Calcott B, Ho SY, Guindon S. (2012). PartitionFinder: combined selection of partitioning schemes and substitution models for phylogenetic analyses. Mol Biol Evol 29: 1695–1701.

Edgcomb VP, Roger AJ, Simpson AGB, Kysela DT, Sogin ML. (2001). Evolutionary relationships among “jakobid” flagellates as indicated by alpha- and beta-tubulin phylogenies. Mol Biol Evol 18: 514–522.

Medlin L, Elwood HJ, Stickel S, Sogin ML. (1988). The characterization of enzymatically amplified eukaryotic 16S-like rRNA-coding regions. Gene 71: 491–499.

Pánek T, Ptáčková E, Čepicka I. (2014). Survey on diversity of marine/saline anaerobic Heterolobosea (Excavata: Discoba) with description of seven new species. Int J Sys Evol Microbiol 64: 2280-2304.

Yabuki A, Kamikawa R, Ishikawa SA, Kolisko M, Kim E, Tanabe AS, Kume K, Ishida KI, Inagki Y. (2014). *Palpitomonas* *bilix* represents a basal cryptist lineage: insight into the character evolution in Cryptista. Scientific reports 4: 4641.

Yoon HS, Grant J, Tekle YI, Wu M, Chaon BC, Cole JC, Logsdon JM, Patterson DJ, Bhattacharya D, Katz LA. (2008). Broadly sampled multigene trees of eukaryotes. BMC Evol Biol 8: 14.
