# Supplementary material for: Combined Culture-Based and Culture-Independent Approaches Provide Insights into Diversity of Jakobids, an Extremely Plesiomorphic Eukaryotic Lineage
Source: Front Microbiol. 2015 Nov 18;6:1288. doi: 10.3389/fmicb.2015.01288 (PMC4649034; doi:10.3389/fmicb.2015.01288)
Supplement: Supplementary file 2 [file DataSheet2.DOCX]

Supplementary Material S2 – Results and Discussion

**Combined culture-based and culture-independent approaches provide insights into diversity of jakobids, extremely plesiomorphic eukaryotic lineage**

Tomáš Pánek^*^, Petr Táborský, Maria G. Pachiadaki, Miluše Hroudová, Čestmír Vlček, Virginia P. Edgcomb & Ivan Čepička

***Correspondence:** Tomáš Pánek, [mistrpanek@seznam.cz](mailto:mistrpanek@seznam.cz)

CONTENTS

[1 Supplementary text 2](#_Toc426726411)

[1.1 Morphology of *Stygiella* spp. (extended) 2](#_Toc426726412)

[1.2 Morphology of *Velundella* spp. (extended) 2](#_Toc426726413)

[1.3 Phylogenetic trees 3](#_Toc426726414)

[1.4 List of revealed pyrotags belonging to Stygiellidae 3](#_Toc426726415)

[1.4.1. List of revealed V4 region pyrotags (97OTU) 3](#_Toc426726416)

[1.4.2. List of revealed V9 region pyrotags (_97_OTU) 4](#_Toc426726417)

[1.4.3 List of revealed V6-V8 region pyrotags (97OTU) 6](#_Toc426726418)

[1.5 Formal description of new taxa (extended) 7](#_Toc426726419)

[2 Supplementary tables 10](#_Toc426726420)

[3 Supplementary figures 11](#_Toc426726421)

[4 Supplementary references 17](#_Toc426726422)

# Supplementary text

## Morphology of *Stygiella* spp. (extended)

Fifteen strains presented in this study belonged to the genus *Stygiella* (see Supplementary Table S1.1.). The strains were morphologically similar to each other (Supplementary Fig. S2.1), usually 6 – 9 µm long, crescent-shaped in lateral view, and possessed a broadly open, diamond-shaped ventral groove. The groove occupied entire or almost entire ventral side of the grooved cell and reached its posterior end (Supplementary Fig. S2.1L, U, HA). Grooved cells attached to the substrate either by flagella (mostly by the anterior flagellum - Supplementary Fig. S2.1I, Z, AA) or by the cell body (laterally or dorsally). The mode of attachment was stable within the strains. The swimming cells were often narrowed from the middle to the posterior end (Supplementary Fig. S2.1JA).

Eight strains (COORONG, EVROS1I, EVROS1T, FUEN2, GOUVIA, KALOGRIA, NORMAR, and OROSEI) belonged to the previously described species *Stygiella* *incarcerata* comb. nov. (formerly *Andalucia incarcerata*; Supplementary Fig. S2.1N – AA). The cells were approximately 7.8 μm long and had an ovoid or elongately ovoid shape (in a ventral view). The length of the anterior flagellum of grooved cells did not exceed the body length except for EVROS1I, where the anterior flagellum of some cells was extremely long, more than twice the cell length. The posterior flagellum of *S*. *incarcerata* was 1.5 times longer than the cell body. In all strains but OROSEI the grooved cells most often swum freely and adhered only occasionally; most cells of the strain OROSEI were attached. Grooved cells of *S*. *incarcerata* attached to the substrate by the anterior flagellum. A few cells were observed that were attached to the substrate through the cytoplasmic projections on the posterior end of the cell body (strain GOUVIA). Swimming cells were relatively rare in all examined strains.

Cells of *Stygiella* *adhaerens* sp. nov. (strains COORONG2, PETROCHORI, and LARNAKA2N; Supplementary Figs. S2.1A – I) and *Stygiella* *cryptica* sp. nov. (strain PC1; Supplementary Figs. S1.1J – M) were morphologically identical and very similar to that of *S.* *incarcerata*. *Stygiella* *cryptica* was the only stygiellid that possessed distinct helix E23/3 in the SSU rRNA hypervariable region V4 (see Supplementary Fig. S2.4). The cells were approximately 7.0 µm (*S*. *adhaerens*) or 8.0 µm (*S*. *cryptica*) long. Unlike most *S*. *incarcerata* strains, the grooved cells of both species almost always adhered to the substrate by the anterior or, seldom, posterior flagellum. Swimming cells were rare.

*Stygiella* *agilis* sp. nov. (Supplementary Fig. S2.1BA – RA) was approximately 7.1 μm long. Unlike in other *Stygiella* spp., the grooved cells adhered to the substrate by the cell body (laterally or dorsally) and were somewhat narrower. Grooved cells swam rarely, whereas swimming cells were extremely abundant and constituted the major component of the population (Supplementary Fig. S2.1EA, JA). Sometimes, we observed also cells attached to the substrate by cytoplasmic projections on the posterior end of the body (Supplementary Fig. S2.1OA, PA). The initial step of the attachment of the grooved cells was mediated by the anterior flagellum. Subsequently, cells attached by the cell body and released the anterior flagellum.

## Morphology of *Velundella* spp. (extended)

Cells of *Velundella* gen. nov. were usually 9 – 12 μm long. Grooved cells (Supplementary Fig. S2.2) possessed a conspicuous, more or less spiral feeding groove that did not reach the posterior end of the cell.

*Velundella* *trypanoides* sp. nov. (strains BUSSPRAND, LUC3N, LARNAKA, NORMAR2, and MURANO3; Figs. S1.2A – L) displayed markedly spiral feeding groove that almost reached the posterior end of the cell. The cells were noticeably elongated when compared to *Stygiella* spp. The anterior flagellum was usually shorter than the cell body, and the posterior flagellum was approximately 1.5 – 2 times longer than the cell. Grooved cells were broad and possessed a conspicuous feeding groove, while serpentine swimming cells bore less apparent, narrower groove (Supplementary Fig. S2.2G). Virtually all grooved cells were attached to the substrate by the cell body or, sometimes, by posteriorly located cytoplasmic projections.

Only a single strain of *Velundella* *nauta* sp. nov. was cultured and examined (strain BMAND; Supplementary Fig. S2.2M – T). Both, anterior and posterior flagella were of the same length as in *V. trypanoides*, although the ventral groove was less spiral and somewhat shorter. The posterior pole of the cell sometimes possessed a bulbous protrusion (Supplementary Fig. S2.2O, P). Most grooved cells were attached by flagella, mainly by anterior ones (Fig. S2.2S, T). Swimming cells possessed distinctly shortened, narrow feeding groove (Supplementary Fig. S2.2Q). Unlike the grooved cells, where the posterior flagellum of swimming cells exited the cell at its posterior pole (Supplementary Fig. S2.2N), the posterior flagellum of swimming cells got off the shortened groove approximately in ½ of the cell length (Supplementary Fig. S2.2Q).

When cultured under harsh conditions (higher concentration of oxygen), rounded forms were observed. The form was markedly similar to putative cysts of *Andalucia* *godoyi* (Lara et al., 2006; Supplementary Fig. S2.3). On the other hand, clear cyst wall was observed neither in *A*. *godoyi*, nor in *V*. *trypanoides*.

## Phylogenetic trees

Before we computed final **SSU rDNA tree** (Figure 1), we performed phylogenetic analysis based on all SSU rDNA clones available. This initial dataset contained also clones shorter than 490 bp (see Supplementary Figure S2.5). To increase statistical support, we removed short sequences (<490 bp) from the final dataset.

We performed phylogenetic analysis of the **V9 region** including all available stygiellid sequences (see Supplementary Figure S2.6.). Genus *Stygiella* as well as EC II and IV were highly supported, while statistical support for other species was low. Therefore, we were able to determine species affiliation of 233 pyrotags belonging to EC II or IV (five OTU_97_) and 97 pyrotags of *Stygiella* spp. (four OTU_97_).

**The alpha-tubulin gene** was not included in the final multi-protein analysis, because Andalucina and Histionina have strikingly different sequences that possibly represent different paralogues (see Supplementary Fig. S2.7.).

## List of revealed pyrotags belonging to Stygiellidae

## 1.4.1. List of revealed V4 region pyrotags (97OTU)

Sequences from Stoeck *et al*., 2010

**>EC_I STOECK831 1 sequence**

CCAGCAGCTGCGGTAATTCCAGCTCCAATAGCGTATATTAAAGTTGTTGCAGTTAAAAAGCTCGTAGTTGGATTTCGGAACGGCGCAGCGGGTCCCAGACGGTTTGTTTGGCGCACCCGCTGTTTTTAGCCGTCCCTAAGGTCGGACTCCCGTGGGGCTCCGCTTCCTCATCGGTTGCGGGCCCCACACTCAGGGCATTCGACCACGTTACTGTGAAAAAATCAGAGTGTTCAAAGCAGGCGCTCGCCTTGAATGGATTAGCATGGAATTACA

## 1.4.2. List of revealed V9 region pyrotags (_97_OTU)

Sequences from Stoeck *et al*., 2009 and Edgcomb *et al*., 2011

**>S_incarcerata STOECK2265 4 sequences**

ccctgccttttgtacacaccgcccgtcgctcctaccgatggatggtccggtgaaatcttcggatgttctttaatctttttcactcgttggaaaagttagagagcagaagttgattaaactttatcatctagaggaaggagaagtcgtgacaaggtttccgtaggtgaacctgcggaagg

**>S_incarcerata STOECK3347 5 sequences**

ccctgccctttgtaccacaccgcccgtcgctcctaccgatggatggtccggtgaaatcttcggatgttctttaatctttttcactcgttggaaaagttagagatcagaagttgattaaactttatcatctagaggaaggagaagtcgtaacaaggtttccgtaggtgaacctgcagaagg

**>S_incarcerata STOECK9680 24 sequences**

ttgtacacaccgcccgtcgctcctaccgatggatggtccggtgaaatcttcggatgttctttaatctttttcactcgttggaagagttagagagcagaagttgattaaactttatcatctagaggaaggagaagtcgtaacaaggtttccgtaggtgaacctgcagaagg

**>S_incarcerata CARIACO11 64 sequences**

gtcgctcctaccgatggatggtccggtgaaatcttcggatgttctttaatctttttcactcgttggaagagttagagagcagaagttgattaaactttatcatctagaggaaggagaagtcgtaacaaggtttcc

**>V_trypanoides CARIACO2918 4 sequences**

gtcgctcctaccgattggatggtccggtgaaatcttcggatcgtgcactctggctggttttttcctgtcatgagcgcgcgagaagttgattaaactttatcatctagaggaaggagaagtcgtaacaaggtttcc

**>V_trypanoides STOECK10922 2 sequences**

ccctgccctttgtacacaccgcccgtcgctcctaccgattggatggtccggtgaaatcttcggatcgtgcactctggctggttttttcctgtcatgagcgcgcgagaagttgattaaactttatcatctagaggaaggagaagtcgtaacaaggtttccgtaggtgaacctgcagaagg

**>EC_IV STOECK9689 30 sequences**

TTGTACACACCGCCCGTCGCTCCTACCGATTGGATGGTCCGGTGAAATCTTCGGATCGCTCCTGCAAGCGGGTTCTCGAACTTGACAAGCGGAGCGAGAAGTTGATTAAACTTTATCATCTAGAGGAAGGAGAAGTCGTAACAAGGTTTCCGTAGGTGAACCTGCAGAAGG

**>EC_IV CARIACO837 52 sequences**

gtcgctcctaccgattggatggtccggtgaaatcttcggatcgctcctgcaagcgggttctcgaacttgacaagcggagcgagaagttgattaaactttatcatctagaggaaggagaagtcgtaacaaggtttcc

**>EC_II CARIACO3704 100 sequences**

gtcgctcctaccgattggatggtccggtgaaatctccggatcgtgcgaactttctggtttaccagagttttgtttgcatgagaagttgattaaactttatcatctagaggaaggagaagtcgtaacaaggtttcc

**>EC_II STOECK8770 47 sequences**

ttgtacacaccgcccgtcgctcctaccgattggatggtccggtgaaatctccggatcgtgcgaactttctggtttaccagagttttgtttgcatgagaagttgattaaactttatcatctagaggaaggagaagtcgtaacaaggtttccgtaggtgaacctgcagaagg

**>EC_II STOECK9295 4 sequences**

ccctgccttttgtacacaccgcccgtcgctcctaccgattggatggtccggtgaaatctccggatcgtgcgaactttctggtttaccagagttttgtttgcatgagaagttgattaaactttatcatctaggggaaggagaagtcgtaacaaggtttccgtaggtgaacctgcagaagg

**>EC_V STOECK5138 1 sequence**

ccctgccctttgtacacaccgcccgtcgcccctaccgattggatgatccggtgaaatcttcggatcgacgcgcacgctcggccctcgggtcctgtcgcgcgcgccgagaagttgattaaactttatcatctagaggaaggagaagtcgtaacaaggtttccgtaggtgaacctgcagaagg

**>EC_V STOECK9483 4 sequences**

ttgtacacaccgcccgtcgctcctaccgattggatgatccggtgaaatcttcggatcgacgcgcacgctcggccctcgggtcctgtcgcgcgcgccgagaagttgattaaactttatcatctagaggaaggagaagtcgtaacaaggtttccgtaggtgaacctgcggaagg

**>EC_V STOECK932 11 sequences**

ttgtacacaccgcccgtcgctcctaccgattggatgatccggtgaaatcttcggatcgacgcgcacgctcggccctcgggtcctgtcgcgcgcgccgagaagttgattaaactttatcatctagaggaaggagaagtcgtaacaaggtttccgtaggtgaacctgcgaagg

**>EC_V CARIACO4688 10 sequences**

GTCGCTCCTACCGATTGGATGATCCGGTGAAATCTTCGGATCGACGCGCACGCTCGGCCCTCGGGTCCTGTCGCGCGCGCCGAGAAGTTGATTAAACTTTATCATCTAGAGGAAGGAGAAGTCGTAACAAGGTTTCC

**>EC_I CARIACO5249 136 sequences**

gtcgctcctaccgattggatgatccggtgaagtcttcggatcgacgtgcccgcgagccttcgggctttggtcgagcgcgacgagaagttgattaaactttatcatctagaggaaggagaagtcgtaacaaggtttcc

**>EC_I STOECK4283 1 sequence**

ccctgccatttgtacacaccgcccgtcgctcctaccgattggatgatccggtgaagtcttcggatcgacgtgcccgcgagccttcgggctttggtcgagcgcgacgagaagttgattaaactttatcatctagaggaaggagaagtcgtaacaaggtttccgtaggtgaacctgcagaagg

**>EC_I STOECK6969 64 sequences**

ccctgccatttgtacacaccgcccgtcgctcctaccgattggatgatccggtgaagtcttcggatcgacgtgcccgcgagccttcgggctttggtcgagcgcgacgagaagttgattaaactttatcatctagaggaaggagaagtcgtaacaaggtttccgtaggtgaacctgcagaagg

**>EC_I STOECK6295 2 sequences**

ttgaacacaccgcccgtcgctcctaccgattggatgatccggtgaagtcttcggatcgacgtgcccgcgagccttcgggctttggtcgagcgcgacgagaagttgattaaactttatcatctagaggaaggagaagtcgtaacaaggtttccgtaggtgaacctgcggaagg

**>EC_I STOECK9147 1 sequence**

ttgtacacaccgcccgtcgctcctaccgattggatgatccggtgaagtcttcggatcgacgtgcccgcgagccttcgggctttggtcgagcgcgacgagaagttgattaaactttatcatctagaggaaggagaagtcgtaacaaggtttccgtaggtgaacctgcgaagg

**>EC_I STOECK543 3 sequences**

ttgtacacaccgcccgtcgctcctaccgattggatgatccggtgaagtcttcggatcgacgtgcccgcgagccttcgggctttggccgagcgcgacgagaagttgattaaactttatcatctagaggaaggagaagtcgtaacaaggtttccgtaggtgaacctgcagaagg

**>EC_I CARIACO3606 2 sequences**

GTCGCTCCTACCGATTGGATGATCCGGTGAAGTCTTCGGATCGACGTGCACGCGAGCCTCCGGGCTTTTGCCGAGCACGACGAGAAGTTGATTAAACTTTATCATCTAGAGGAAGGAGTAGTCGTAACAAGGTTTCC

**>EC_I STOECK10839 1 sequence**

ccctgccctttgtacacaccgcccgtcgctcctaccgattggatgatccggtgaagtcttcggatcgacgtgcacgcgagcctccgggcttttgccgagcacgacgagaagttgattaaactttatcatctagaggaaggagtagtcgtaacaaggtttccgtaggtgaacctgcagaagg

## 1.4.3 List of revealed V6-V8 region pyrotags (97OTU)

Sequence provided by S. Hallam (BioProject PRJNA247822)

>**EC_IV 3198Saanitch** **56 sequences**

AAAGGGCAGGGACGTATTCAGCGCGAGTTGATGACTCGCGCTTACAAGGAATTCCTCGTTGAAGACCGATAATTGCAAAGGTCTATCCCCATCACGACGGACGCTCCGAGATTACCCAGACCTTCCGGTCGAGGAAAAACTAGTTGTGTCCGTCAGTGTAGCGCGCGTGCAGCCCAGGACATCTAAGGGCATCACAGACCTGTTATTGCCTCAAACTTCCATGCGTTAAACACGCATAGTCCCTCTAAGAAGTTGGAAAGTTCGATTCCGAAAAGGAATCGTCCAACTATTTAGTAGGTTAAGGTCTCGTTCGTTAACGGAATTAACCAGACAAATCACTCCACCAACTAAGAACGGCCATGCACCACCACCCATAGAATCAAGAAAGAGCTATCAATCTGTCCATCCTAACTATGTCCTGACCTGGTAAGTTTCCCCGTGTTGAGTCAAATTAAGCCGCAGGCTCCACTCCTGGTGGTG

## Formal description of new taxa (extended)

Excavata: Discoba: Jakobida: Andalucina

**Stygiellidae fam. nov. Pánek, Táborský** & **Čepička**.

Diagnosis: Aloricate marine jakobids with acristate mitochondria.

Type genus: *Stygiella* Pánek, Táborský & Čepička, 2014.

Other genus: *Velundella* gen. nov. Pánek, Táborský & Čepička, 2014.

Zoobank registration: urn:lsid:zoobank.org:act:4A238129-B037-4B4E-9D15-E205E7B13605.

***Stygiella* gen. nov. Pánek, Táborský** & **Čepička**.

Diagnosis: Stygiellidae with grooved cells usually 6–9 µm long, crescent-shaped (in lateral view), with a broadly open, diamond-shaped feeding groove. The groove occupies entire or almost entire ventral side of the grooved cell and reached its posterior end.

Type species: *Jakoba* *incarcerata* Bernard Simpson & Patterson, 2000 (= *Andalucia incarcerata*, *Stygiella incarcerata*).

Etymology: N.L. fem. dim. n. *Stygiella*, named after the goddess of the river Styx. This river formed a boundary between the Earth and the Underworld.

Zoobank registration: urn:lsid:zoobank.org:act:9EA9ADF7-11C7-40D7-925F-7845D48420C0.

***Stygiella* *incarcerata* (Bernard, Simpson & Patterson, 2000) comb. nov.**

Synonyms: *Jakoba incarcerata* Bernard, Simpson & Patterson, 2000; *Andalucia incarcerata* (Bernard, Simpson & Patterson, 2000).

Description: grooved cells 6.7–9.5 µm long, often attached to the substrate by the anterior flagellum. Swimming cells relatively rare.

***Stygiella adhaerens* sp. nov. Pánek, Táborský** & **Čepička**.

Diagnosis: *Stygiella* with grooved cells 5.0–8.4 µm long. Grooved cells very often adhered to the substrate by the anterior or the posterior flagellum and swam rarely.

Syntype: Protargol preparation of the isolate PETROCHORI, deposited in the collection of the Department of Parasitology, Faculty of Science, Charles University, Prague, Czech Republic, catalogue number 12/96. Fig. 1Z, AA is image from the syntype.

Type locality: Pilos, Petrochori, Greece (36^o^58’N, 21^o^39’E).

Habitat: Anoxic marine sediment.

Etymology: L. part. adj. *adhaerens* (adhering).

Gene sequence: SSU rDNA sequence from the strain PETROCHORI, GenBank accession number KP144401.

Zoobank registration: urn:lsid:zoobank.org:act:E2EF3439-3683-42F1-8985-5A550683F374.

***Stygiella agilis* sp. nov. Pánek, Táborský & Čepička.**

Diagnosis: *Stygiella* with grooved cells 5.6–8.9 µm long. Grooved cells adhered to the substrate by the cell body (laterally or dorsally), swam rarely. Swimming cells were extremely abundant and constituted dominant cell morphotype in the population.

Syntype: Protargol preparations of the isolate AND, deposited in the collection of the Dept. of Parasitology, Faculty of Science, Charles University, Prague, Czech Republic, catalogue numbers 9/30-32 and 10/99, 100. Fig. 1AC is image from the syntype.

Type locality: Elephant beach, Havelock Island, Andaman Islands, India (12^o^00’N, 92^o^56’E).

Habitat: Anoxic marine sediment.

Etymology: L. adj. *agilis* (agile).

Gene sequence: SSU rDNA sequence from the strain AND, GenBank accession number KP144396.

Zoobank registration: urn:lsid:zoobank.org:act:A0114EE7-55D7-4960-8B92-E1AC20019774.

***Stygiella cryptica* sp. nov. Pánek, Táborský** & **Čepička**.

Diagnosis: *Stygiella* with grooved cells 6.4–10.5 µm long. Grooved cells very often adhered to the substrate by the anterior or the posterior flagellum and swam rarely. SSU rRNA possesses distinct helix E23/3 in the hypervariable region V4.

Syntype: Protargol preparations of the isolate PC1, deposited in the collection of the Department of Parasitology, Faculty of Science, Charles University, Prague, Czech Republic, catalogue numbers 5/97 and 6/51, 52. Fig. 1X, Y is image from the syntype.

Type locality: Peggy’s Cove, Canada (44^o^29’N, 63^o^55’W).

Habitat: Anoxic marine sediment.

Etymology: L. adj. *cryptica* (cryptic).

Gene sequence: SSU rDNA sequence from the strain PC1, GenBank accession number KP144402.

Zoobank registration: urn:lsid:zoobank.org:act:21C5B523-3A4E-405C-A7B4-6507E31F3C1B.

***Velundella* gen. nov. Pánek, Táborský** & **Čepička.**

Diagnosis: Stygiellidae usually 9–12 μm long. Grooved cells possessed a distinct, spiral feeding groove that did not reach the posterior end of the cell.

Type species: *Velundella trypanoides* sp. nov. Pánek, Táborský & Čepička.

Etymology: N.L. fem. dim. n. *Velundella*, named after the Velvet Underground, an American rock band that represent underground music and is often considered as one of the most important and influential groups of the 1960s.

Zoobank registration: urn:lsid:zoobank.org:act:9472415A-259E-484C-83B2-E0D3B273CD32.

***Velundella trypanoides* sp. nov. Pánek, Táborský & Čepička.**

Diagnosis: *Velundella* 7.8–14.9 µm long, a markedly spiral feeding groove almost reaches the posterior end of the cell. Cells noticeably elongated. Grooved cells broad, with conspicuous groove; groove of serpentine-shaped swimming cells is less apparent and narrower. Virtually all grooved cells attached to the substrate by the cell body or, sometimes, by posterior cytoplasmic projections.

Syntype: Protargol preparations of the strain LUC3N, deposited in the collection of the Department of Parasitology, Faculty of Science, Charles University, Prague, Czech Republic, catalogue numbers 6/27, 28, 84, 85. Fig. 1AD, AE are images from the syntype.

Type locality: Brač island, Croatia (43^o^17’N, 16^o^52’E).

Habitat: Anoxic marine sediment.

Etymology: derived from Gr. n. *trypáni* (auger, drill) and L. suffix -*oides* (resembling, similar).

Gene sequence: SSU rDNA sequence from the strain LUC3N, GenBank accession number KP144395.

Zoobank registration: urn:lsid:zoobank.org:act:3879E18C-D7F9-4D48-B0B4-EEF635D39664.

***Velundella nauta* sp. nov. Pánek, Táborský** & **Čepička**.

Diagnosis: *Velundella* with grooved cells 8.4–11.8 µm long; less spiral and shorter groove than *V. trypanoides*. The majority of grooved cells attached to the substrate by flagella, mainly by the anterior one; bulbous protrusion at the posterior pole of the cell. The posterior flagellum of the swimming cells leaves the shortened groove approximately in ½ of the cell length.

Syntype: Protargol preparations of the strain BMT, deposited in the collection of the Department of Parasitology, Faculty of Science, Charles University, Prague, Czech Republic, catalogue numbers 9/92, 93. Fig. 1AF, AG is image from the syntype.

Type locality: Balikpapan, Kalimantan, Indonesia (1°20' S, 116°50' E).

Habitat: Anoxic marine sediment.

Etymology: L. masc. n. *nauta* (a sailor).

Gene sequence: SSU rDNA sequence from the strain LUC3N, GenBank accession number KP144392.

Zoobank registration: urn:lsid:zoobank.org:act:3879E18C-D7F9-4D48-B0B4-EEF635D39664.

# Supplementary tables

**Supplementary Table S2.1. Dimensions (in μm) of living and protargol stained specimens of *Stygiella* and *Velundella* strains and species: average of cell lengths ± standard deviation (range).** CL – cell length, LIV – living cells, n – number of cells, PTG – protargol-stained cells.

| **Strain / species** | **CL LIV** | **n** | **CL PTG** | **n** |
| --- | --- | --- | --- | --- |
| ***Stygiella incarcerata* comb. nov. (= *Andalucia* *incarcerata*)** | | | | |
| EVROS1I | 7.7 ± 0.4 (7.1 – 8.5) µm | 20 | 4.2 ± 0.5 (3.0 – 4.7) µm | 25 |
| FUEN2 | 7.4 ± 0.5 (7.4 – 9.0) µm | 50 | N.A. | 0 |
| NORMAR | 8.2 ± 0.7 (6.7 – 9.5) µm | 50 | 3.9 ± 0.4 (3.2 – 4.7) µm | 25 |
| OROSEI2A | 7.8 ± 0.5 (6.9 – 9.1) µm | 50 | N.A. | 0 |
| *S. incarcerata* | 7.8 ± 0.6 (6.7 – 9.5) µm | 170 | 4.0 ± 0.5 (3.0 – 4.7) µm | 50 |
| ***Stygiella adhaerens sp. nov.*** | | | | |
| COORONG2 | 7.2 ± 0.7 (6.0 – 8.2) µm | 50 | 4.2 ± 0.4 (3.4 – 5.2) µm | 50 |
| LARNAKA2N | 6.2 ± 0.7 (5.0 – 8.4) µm | 50 | N.A. | 0 |
| PETROCHORI | 7.4 ± 0.5 (6.3 – 8.3) µm | 50 | N.A. | 0 |
| *S. adhaerens* | 7.0 ± 0.8 (5.0 – 8.4) µm | 150 | 4.2 ± 0.5 (3.1 – 5.6) µm | 50 |
| ***Stygiella cryptica* sp. nov.** | | | | |
| PC1 | 8.0 ± 0.8 (6.4 – 10.5) µm | 50 | 4.3 ± 0.6 (3.1 – 5.6) µm | 50 |
| ***Stygiella agilis* sp. nov.** | | | | |
| AND | 7.4 ± 0.8 (6.1 – 9.0) µm | 50 | 3.9 ± 0.5 (3.3 – 4.9) µm | 20 |
| IGO3 | 7.0 ± 0.8 (5.6 – 8.3) µm | 50 | N.A. | 0 |
| MANG | 7.2 ± 0.6 (5.9 – 8.2) µm | 50 | 3.9 ± 0.5 (3.2 – 4.7) µm | 20 |
| *S. agilis* | 7.1 ± 0.7 (5.6 – 8.9) µm | 150 | 3.9 ± 0.5 (3.1 – 4.9) µm | 40 |
| ***Velundella nauta* sp. nov.** | | | | |
| BMAND | 10.1 ± 0.9 (8.4 – 11.8) µm | 50 | 6.3 ± 1.0 (5.1 – 8.1) µm | 27 |
| ***Velundella trypanoides* sp. nov.** | | | | |
| BUSSPRAND | 10.1 ± 0.5 (9.3 – 11.9) µm | 50 | 6.6 ± 0.7 (4.4 – 7.5) µm | 50 |
| LARNAKA | 11.2 ± 1.2 (9.6 – 14.9) µm | 50 | 6.6 ± 0.8 (5.2 – 9.6) µm | 50 |
| LUC3N | 11.1 ± 1.1 (9.5 – 14.1) µm | 50 | 7.2 ± 1.0 (5.8 – 10.6) µm | 50 |
| MURANO3 | 10.7 ± 1.4 (8.8 – 14.6) µm | 50 | N.A. | 0 |
| *V. trypanoides* | 10.4 ± 1.2 (7.8 – 14.9) µm | 200 | 6.5 ± 1.1 (4.4 – 10.6) µm | 150 |

# Supplementary figures

**Supplementary Figure S2.1.** **Living flagellates of the genus *Stygiella* gen. nov.** **observed using Differential Interference Contrast**. Most cells presented here are grooved cells, only a few cells are swimming cells (EA, JA). Species and strains are arranged as follows: *S*. *adhaerens* strain PETROCHORI (A – D) and COORONG2 (E – I); *S. cryptica* strain PC1 (J – M); *S. incarcerata* strain NORMAR (N – R), OROSEI (S – Y) and GOUVIA (Z – AA); *S.* *agillis* strain MANG (BA – EA), AND (FA – JA) and IGO3 (KA – RA). Bar = 5 µm. Arrow: anterior flagellum attached to the substrate; double-arrow: cytoplasmic projections or pseudopodia on cell posterior.

**
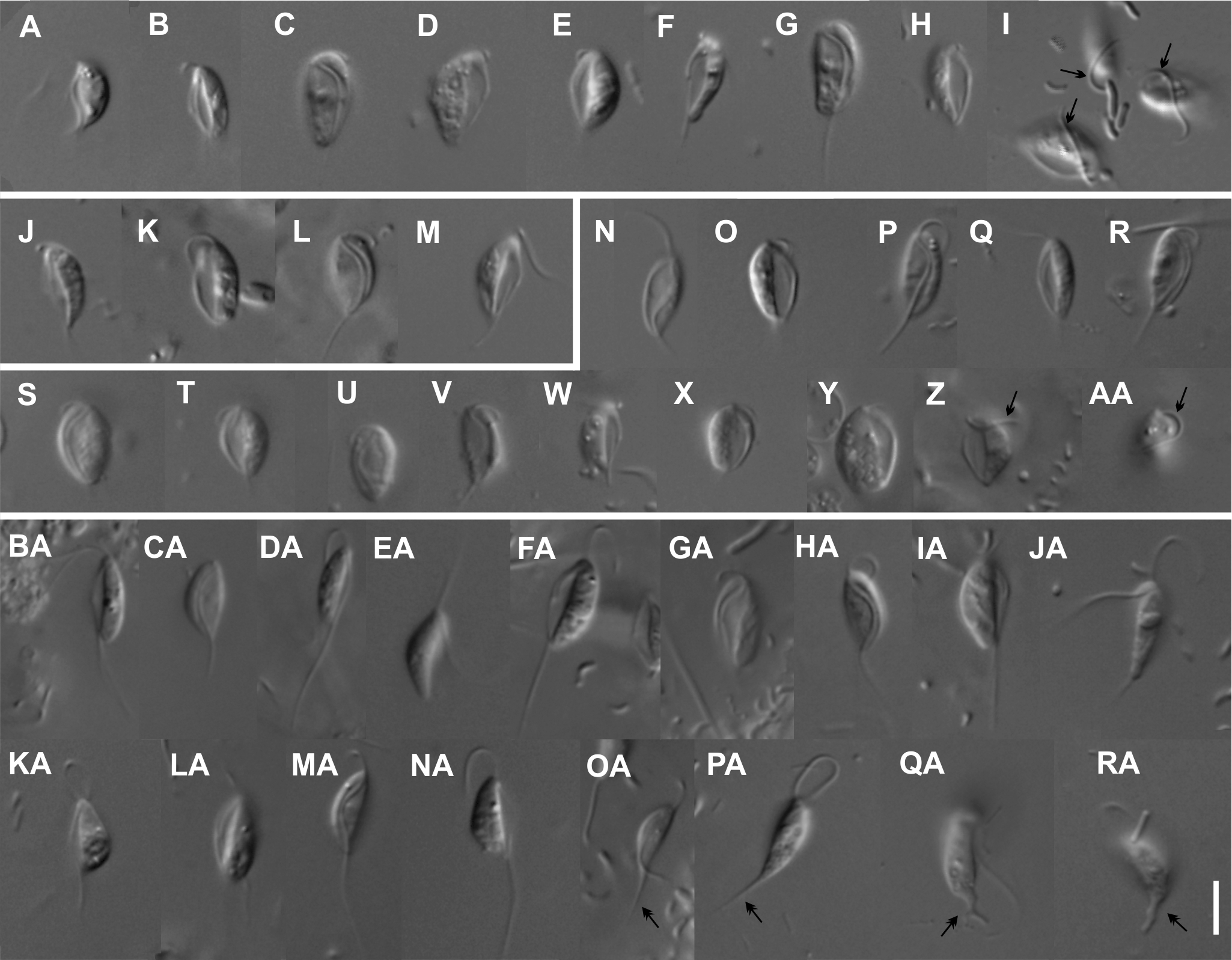
**

**Supplementary Figure S2.2.** **Living flagellates of the genus *Velundella* observed using Differential Interference Contrast**. Most cells presented here are grooved cells, only a few cells are swimming cells (G, Q). Species and strains are arranged as follows: *V*. *trypanoides* strain LUC3N (A – C), BUSSPRAND (D – G), LARNAKA (H – J), MURANO3 (K – L); *V. nauta* strain BMAND (M – T). DIC. Bar = 5 µm. Arrow: anterior flagellum attached to the substrate; double-arrow: a bulbous protrusion on cell posterior of *V*. *nauta*.


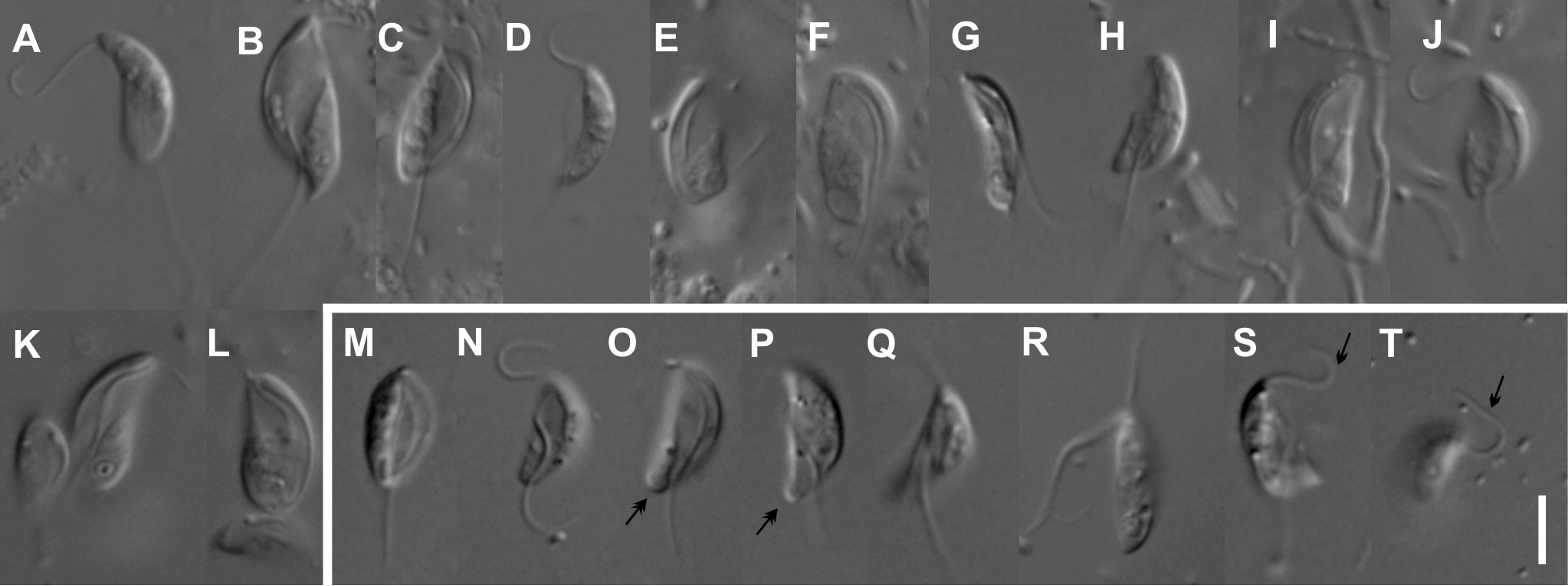


**Supplementary Figure S2.3. Rounded form of *Velundella* *trypanoides* (strain BUSS PRAND).** Cells were observed using DIC.


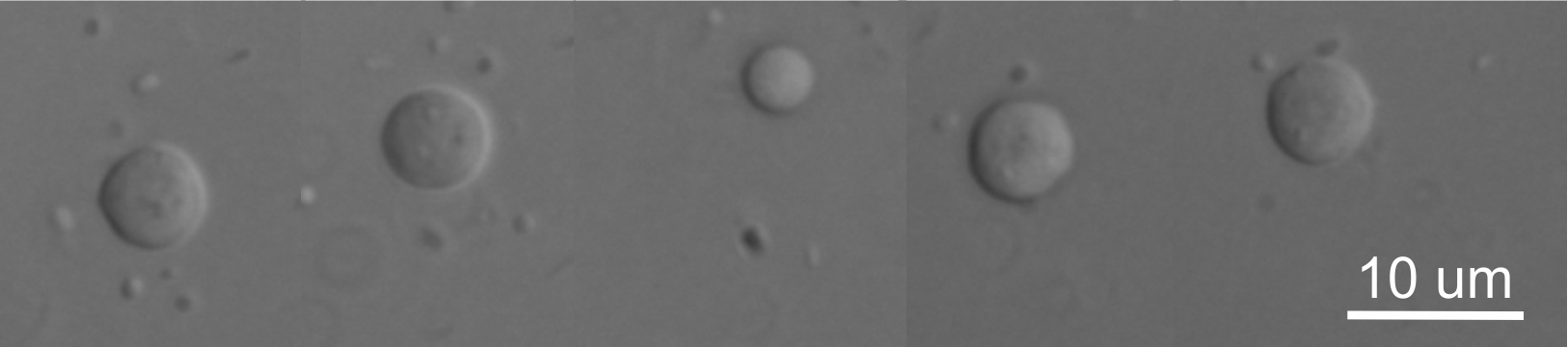


**Supplementary Figure S2.4 Secondary structure of helices e23/1-3 in SSU rRNA (positions 624-738 in the SSU rRNA of *Stygiella* *cryptica* strain PC1). Representative sequences from all described species and environmental clades are documented.** *Velundella* *nauta* strain BMT (A); *V.* *trypanoides* strain LUC3N (B); environmental clade II, DQ310295 (C); environmental clade III, EU446381 (D); *Stygiella* *agilis* strain AND (E); *S.* *adhaerens* strain PETROCHORI (F); *S.* *incarcerata* strain FUEN2 (G); environmental clade IV, FJ153665 (H); *S.* *cryptica* strain PC1 (I); environmental clade I, EF526837 (J). Structures were reconstructed using MFOLD web server (Zucker, 2003) and VARNA 3.91 software.


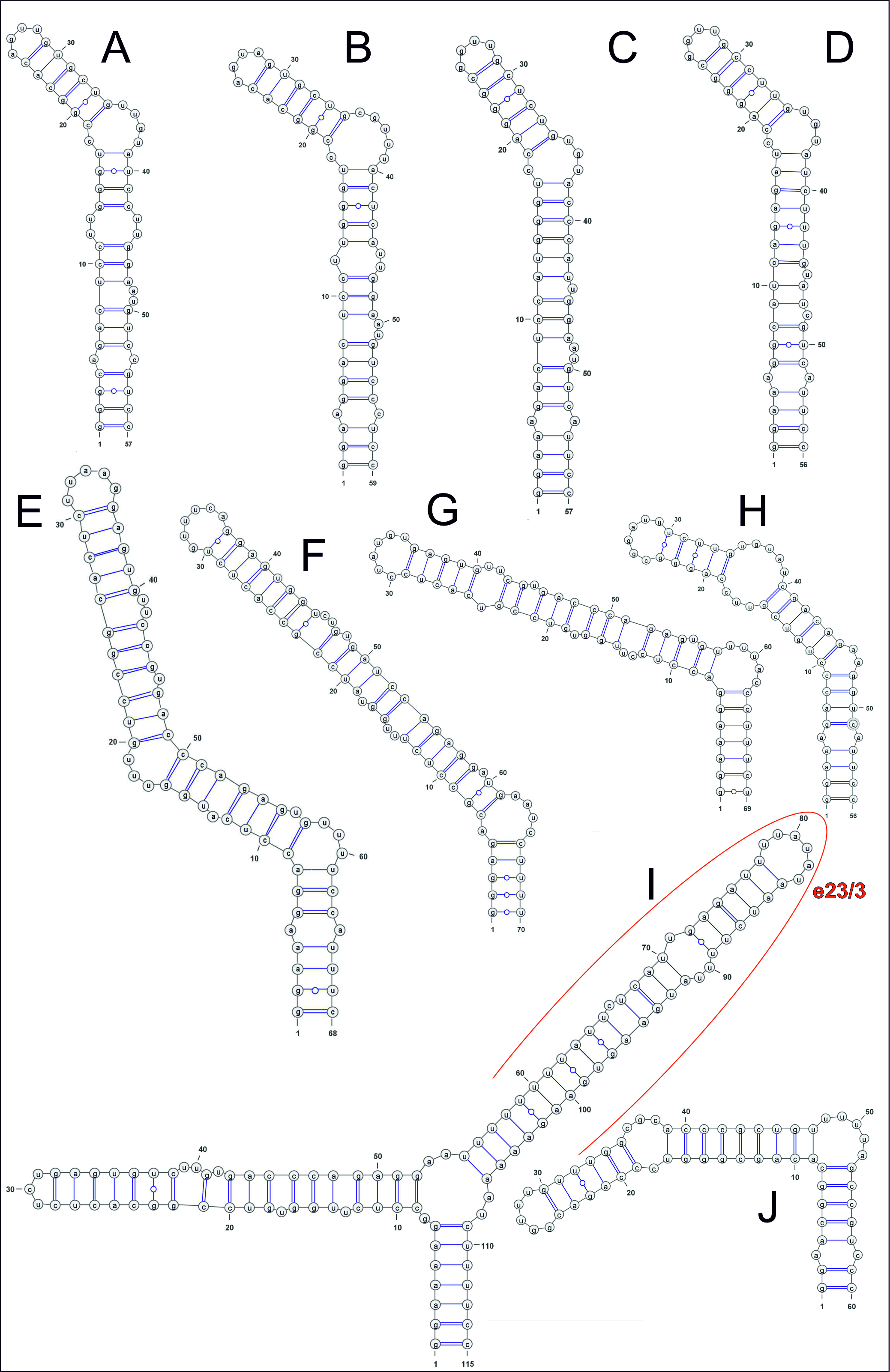


**Supplementary Figure S2.5. Preliminary phylogenetic tree of Stygiellidae based on small subunit ribosomal rRNA (environmental Sanger clones shorter than 490 bp are included).** The tree is based on alignment of 1604 positions and 114 OTUs representing all environmental clones of Stygiellidae. Eight andaluciids and four histionids were used as outgroups. The topology was constructed in RAxML using maximum likelihood (GTRGAMMAI model). The values at nodes represent RAxML bootstraps. Sequences from newly isolated strains are in bold. Environmental sequences are represented by their GenBank accession numbers only.


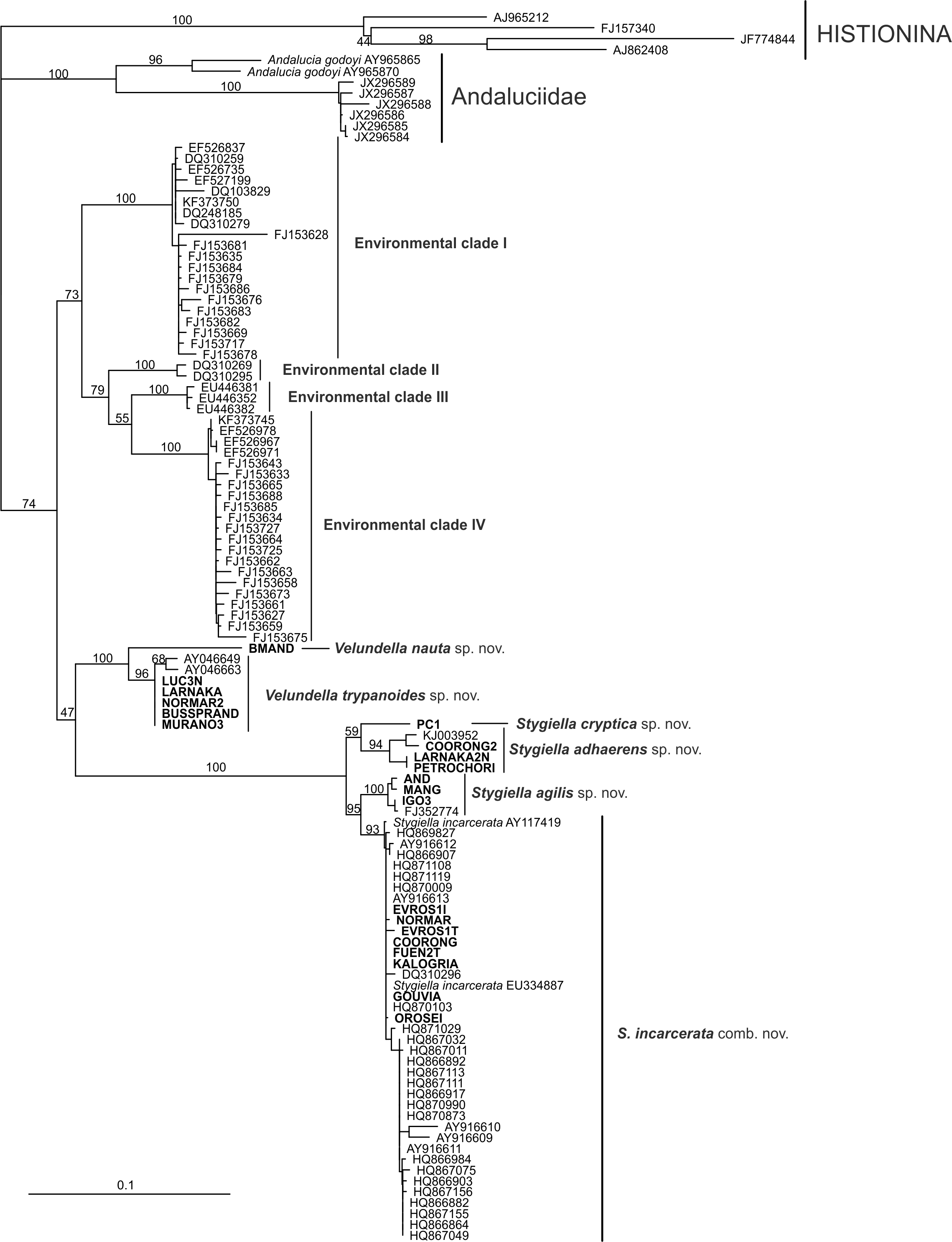


**Supplementary Figure S2.6. Phylogenetic tree of V9 region (Stygiellidae only).** The tree is based on alignment of 263 nucleotides and 57 OTUs. The topology was constructed in RAxML using maximum likelihood (GTRGAMMA model). The values at nodes represent RAxML bootstraps (1000 non-parametric bootstraps were computed). The values lower than 50% are not marked. Sequences (_97_OTUs) from short read archives are in bold.


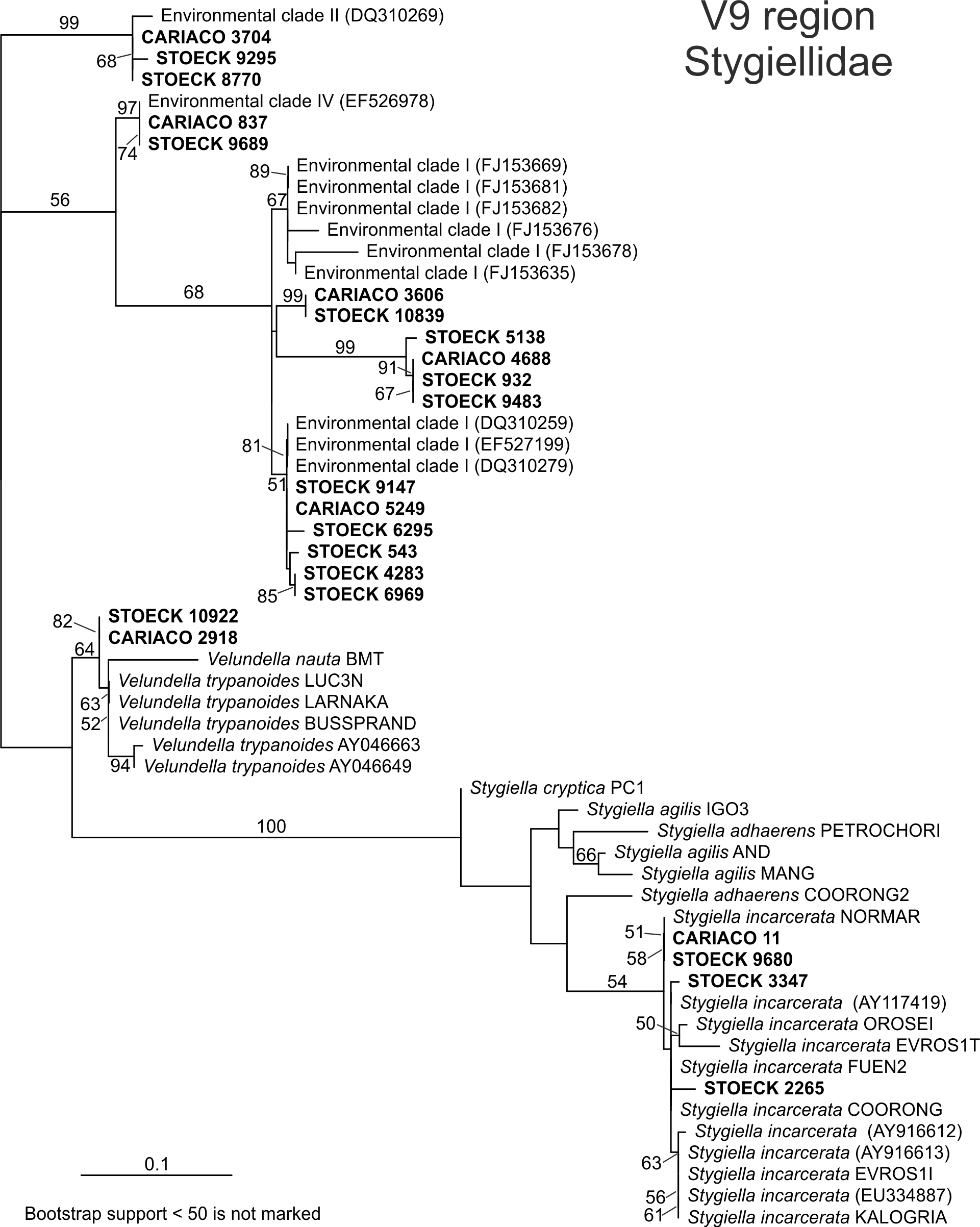


**Supplementary Figure S2.7. Phylogenetic tree of eukaryotes based on α-tubulin. The tree is based on alignment of 405 amino acid residues and 51 taxa.** The topology was constructed in RAxML using maximum likelihood (PROTGAMMAILGF model). PhyloBayes was run under POI CAT model. The values at nodes represent RAxML bootstraps/PhyloBayes posterior probabilities. The values lower than 50% or 0.5 are marked by „-“.Sequences from newly isolated strains are in bold.


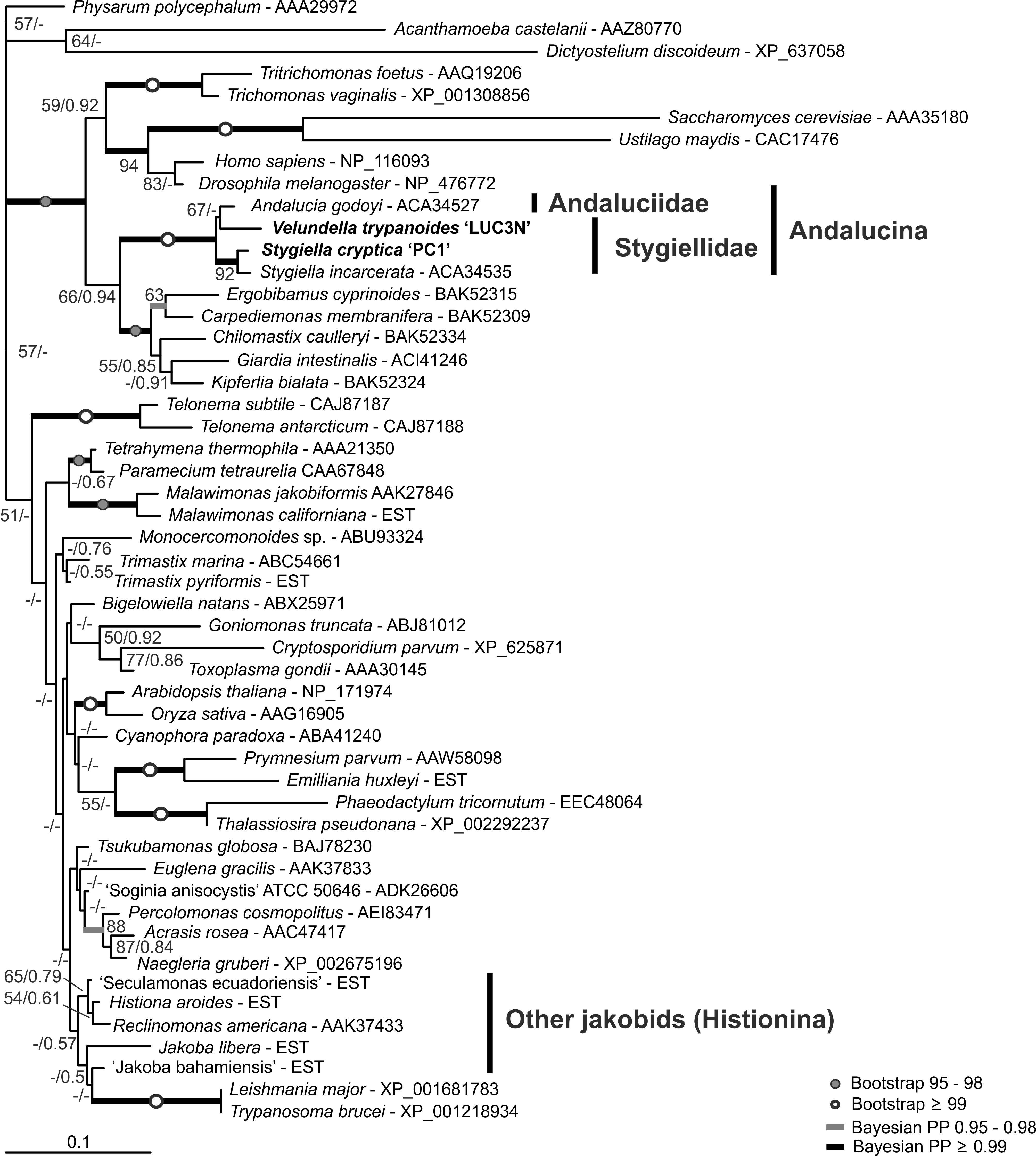


# Supplementary references

Lara E, Chatzinotas A, Simpson AGB. (2006). *Andalucia* (n. gen.) – the deepest branch within jakobids (Jakobida: Excavata), based on morphological and molecular study of a new flagellate from soil. J Eukaryot Microbiol 53: 112–120.

Zuker M. (2003). Mfold web server for nucleic acid folding and hybridization prediction. Nucleic Acids Res. 31: 3406-3415.
